# Metabolic plasticity in synthetic lethal mutants: Viability at higher cost

**DOI:** 10.1371/journal.pcbi.1005949

**Published:** 2018-01-30

**Authors:** Francesco Alessandro Massucci, Francesc Sagués, M. Ángeles Serrano

**Affiliations:** 1 Departament de Física de la Matèria Condensada, Universitat de Barcelona, Martí i Franquès 1, Barcelona, Spain; 2 Departament de Química Física, Universitat de Barcelona, Martí i Franquès 1, Barcelona, Spain; 3 Universitat de Barcelona Institute of Complex Systems (UBICS), Universitat de Barcelona, Barcelona, Spain; 4 ICREA, Pg. Lluís Companys 23, Barcelona, Spain; The Pennsylvania State University, UNITED STATES

## Abstract

The most frequent form of pairwise synthetic lethality (SL) in metabolic networks is known as plasticity synthetic lethality. It occurs when the simultaneous inhibition of paired functional and silent metabolic reactions or genes is lethal, while the default of the functional partner is backed up by the activation of the silent one. Using computational techniques on bacterial genome-scale metabolic reconstructions, we found that the failure of the functional partner triggers a critical reorganization of fluxes to ensure viability in the mutant which not only affects the SL pair but a significant fraction of other interconnected reactions, forming what we call a SL cluster. Interestingly, SL clusters show a strong entanglement both in terms of reactions and genes. This strong overlap mitigates the acquired vulnerabilities and increased structural and functional costs that pay for the robustness provided by essential plasticity. Finally, the participation of coessential reactions and genes in different SL clusters is very heterogeneous and those at the intersection of many SL clusters could serve as supertargets for more efficient drug action in the treatment of complex diseases and to elucidate improved strategies directed to reduce undesired resistance to chemicals in pathogens.

## Author summary

Synthetic lethality (SL), in which the combined knockout of two nonessential genes or reactions is lethal, has direct applications in recognising targets for therapeutic treatment of complex diseases and for fighting against undesired resistance. Typically, SL interactions are reported in pairs. We propose a change of paradigm based on the fact that SL interactions in metabolism are not independent of each other but form complex backup systems involving the rearrangement of a significant SL cluster of metabolic fluxes to ensure viability. This robustness comes at the expenses of acquired vulnerabilities and increased costs, mitigated by the entanglement of SL clusters in terms of shared reactions and genes, which could serve as supertargets for a new generation of therapeutic treatments.

## Introduction

In metabolic networks, phenotypic responses to mutations that block the activity of nonessential biochemical reactions imply a fast rearrangement of fluxes. This metabolic plasticity, understood as a reorganisation or repair of damage in response to a disruption, is a signal of the robustness of metabolism against perturbations [1]. Further indications of metabolism robustness is provided by experimental results showing that more than 90% of the genes in *Escherichia coli* K-12 are probably not essential, with metabolic genes presenting no exception [2].

However, among the viable mutations, some are critically fragile. If a mutated enzyme-coding gene or a disrupted reaction forms a synthetic lethal (SL) pair with a partner, meaning that their simultaneous deletion becomes lethal for the organism even though the individual removals are not [3–7], metabolic plasticity becomes essential to ensure viability. These synthetic lethalities provide the mutant with new vulnerabilities, exploitable for antimicrobial drug target identification [8] or, in the case of eukarya, for cancer therapy [9].

Due to the complex interconnectivity of metabolic networks [10–12], the critical reorganisation of fluxes in most SL mutants may affect a significant fraction of reactions other than the SL pair. This would be specially relevant for SL interactions formed by a functional or active (non-zero flux) and a silent or inactive (zero flux) reaction in the native state.These interactions are called plasticity synthetic lethal (PSL) pairs, the dominant SL category in *Escherichia coli* [7]. Mutants of PSL interactions, in which the inactive reaction in the pair activates as a backup when the active reaction fails, could be impaired by a metabolic burden which should be compensated by specific metabolic/genetic mechanisms. A parallel situation has been observed, for instance, in antibiotic resistant mutants. A nonspecific metabolic stress leading to a potential fitness cost [13] has been proposed to be compensated for by adjusting metabolism without the need for acquiring compensatory mutations [14].

In the following, we use computational techniques on genome-scale metabolic reconstructions [15] of three bacteria in the *Escherichia* genus to define and characterize the metabolic reorganisation related with essential plasticity in PSL mutants. We quantify the increased structural and functional metabolic burden of essential plasticity, and we explore the mechanisms that buffer against the huge costs expected for sustaining alternative backup mechanisms for all PSL pairs in these organisms.

## Results

We studied three bacteria in the *Enterobacteriaceae* family: *Escherichia coli* (*E. coli*), *Shigella sonnei* (*S. sonnei*), and *Salmonella enterica* (*S. enterica*). *E. coli* is the prototypical model organism for bacteria and, to put the results into perspective, we also included in the study *S. sonnei*, known to present strong similarities with *E. coli* [16, 17], and *S. enterica*, a well differentiated bacterium [18]. The specific strains and reconstructions that we use here are: *E. coli* K-12 MG1655 *i*JO1366 [19], *S. sonnei* Ss046 *i*SSON_1240 [20], and *S. enterica* enterica serovar Typhimurium LT2 STM_v1_0 [21]. We simulated *in silico* metabolic fluxes on each genome-scale reconstruction using Flux Balance Analysis (FBA) [22] with optimization of biomass yield in glucose minimal medium, and assumed viability when the flux through the biomass function was nonzero (see Materials and methods). By applying FBA to mutants defective in single or pairs of reactions, we identified all *in silico* single essential reactions and SL reaction pairs in the organisms, as in [5–7]. We classified synthetic lethal combinations as Plasticity SL (PSL) pairs, with one reaction functional and one silent in the WT, and Redundancy SL (RSL) pairs, with both reactions active [7]. In each species, more than 75% of the pairs are in the PSL class (Table 1). Next, we focus on this category both for their abundance and also for the impact that the failure of the functional PSL reaction has in terms of flux reorganization.

**Table 1:** Metabolic reconstructions considered in our study and statistics in glucose minimal medium (see Methods).

Organism		*E. coli* MG1655	*S. sonnei* Ss046	*S. enterica* LT2
Model		*i*JO1366	*i*SSON_1240	STM_v1_0
Reference		[19]	[20]	[21]
*N*_*R*_	all	2583	2694	2546
	unlocked	2284	2334	2227
	FVA	1520	1757	1678
	active	418	450	498
*N*_*M*_	actual	1136	1216	1119
	synth	1805	1938	1802
Single Essential		289	282	346
Plasticity SL		231	233	109
Redundancy SL		25	30	31
PSL clusters	*R*_*switches*_	60 (79)	63	61
	*R*_*backups*_	59 (136)	60	63
	*R*_*noncoess*_	168 (293)	152	116
	Total *R*	287 (508)	275	240
	Gene units	196	161	155
[*F*^*mutant*^/*F*^*WT*^]_*cluster*_		542.69	6.56	90446.79
*A*^*mutant*^/*A*^*WT*^		1.56	176.14	0.64
$E_{NG}^{mutant}/E_{NG}^{WT}$		15.56	1.25	1.25
*C*_*BgtS*_		0.64	0.64	0.37
*C*_*BgtNC*_		0.56	0.55	0.49
〈*C*_*B*〉_		0.50	0.48	0.58
〈*C*_*S*〉_		0.43	0.44	0.55
〈*C*_*NCE*〉_		0.41	0.40	0.48
p-values K-S test	SL cluster sizes	0.012	0.004	0.202
	Ranking in clusters: S vs B	0.073	0.252	0.289
	Ranking in clusters: S vs NC	0.005	0.007	0.000
	Ranking in clusters: B vs NC	0.004	0.043	0.002

### Definition of SL cluster

We name the WT functional reaction in a PSL pair a metabolic switch, and the WT silent coessential partner its backup. When the metabolic switch fails, phenotypic reorganization takes place to allow viability. More specifically, the inactive reaction in the PSL pair turns on acting as a functional backup which buffers against the mutation. Notice that one switch can have more than one backup, with proportions close to 4 backups for every switch in *E. coli* and *S. sonnei* to 1 in *S. enterica* (Table 1). For the three bacteria, all the backups associated with the same switch activate together, forming what we call a backup system.

**Fig 1:**
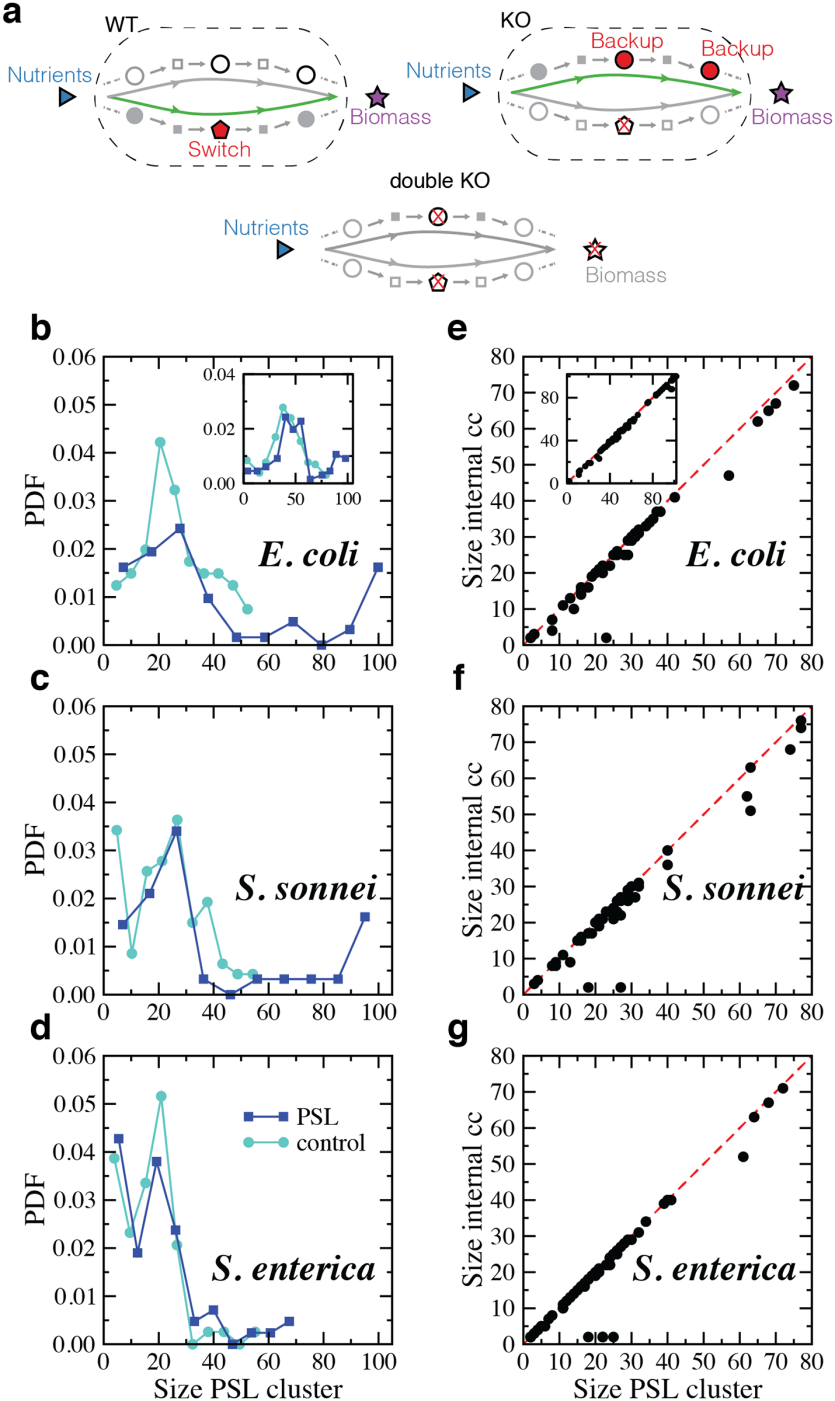
Sketch of a SL cluster and distributions of SL clusters sizes. **a.** In the WT, the metabolic flow happens through a switch reaction (the red pentagon). When it fails, the metabolic flow goes through an alternative channel, that encompasses the backup system of the switch (red circles). When both the switch and one of its backups are deleted, growth may no longer be attained. The SL cluster is denoted by all reactions inside the dashed box. **b-d.** Probability distribution functions of SL clusters sizes for PSL mutants in minimal medium as compared to the control (given by mutants of active, nonessential and noncoessential reactions). **e-g.** Number of reactions in SL clusters as compared to the sizes of the corresponding internal connected components in minimal medium. **Inset e.** The same as in **e** for *E. coli* in rich medium.

At the same time, other fluxes reorganize such that reactions that were inactive in the WT become active in the mutant and vice versa. For every switch of *E. coli*, *S. sonnei* and *S. enterica*, we identified the differentially activated reactions in the mutant which changed from active to inactive or from inactive to active. The whole set is named the SL cluster, (Fig 1a). By definition, the SL cluster contains the switch and its backup system and the switch is unique to a SL cluster and cannot enter as a backup in any other. However, backups can serve more than one switch, and both switches and backups can enter as differentially activated reactions in other SL clusters as well. In the studied organisms, the SL clusters have a small but significant size, comprising groups of about 5% of the total number of reaction in the genome-scale reconstructions. The size distributions for all SL clusters in the three bacteria are shown in (Fig 1b–1d. As a control, we use the differentially activated reaction sets resulting in mutants obtained when reactions which are active in WT but not essential or coessential are knocked out. To discern whether the distributions of observed SL cluster sizes were compatible with the distributions given by the control we calculated the p-value given by the Kolmogorov-Smirnov (K-S) test. The results in Table 1 show that we can reject that the distributions are the same for *E. coli* and *S. sonnei*, since the K-S p-value is well below 10%, while it is not the case for *S. enterica*. The comparison of the curves shows that the size distribution of SL clusters has a long tail in *E. coli* and *S. sonnei* which is absent in the control. Therefore, essential plasticity is more complex and involves more metabolic reactions than reorganizations induced by nonessential mutations. This is also valid for results in rich medium, see inset (Fig 1b).

Reactions in SL clusters form cohesive structures. First, the switch belongs to a connected component of differentially activated reactions which includes its backups The only exception corresponds to the SL cluster associated to the switch reaction phosphoenolpyruvate carboxylase in *S. enterica*; it has Isocitrate lyase as its only backup, which is not included in the internal connected component but forms an isolated component inside the corresponding SL cluster. Second, this connected component is the largest in the SL cluster and on average includes more than 92% of its reactions, (Fig 1e–1g). The rest form residual disconnected components scattered through the metabolic network. Only 2 clusters in *E. coli* and *S. sonnei* and 4 in *S. enterica* out of approximately 60 in each organism (Table 1) deviate from this behavior. The explanation for the divergence of the sizes of the SL cluster and its connected component is most frequently a change of strategy in mutants, like the switch from aerobic to anaerobic metabolism associated with the extracellullar transport of oxygen in *E. coli*, and to the reaction protoporphyrinogen oxidase in *S. sonnei*. Special mention deserves the SL cluster in the three organisms associated with the extracellular transport of glucose via diffusion. Both the switch and the backup are connected to exactly the same reactants and products. Therefore, they are equivalent alternatives for FBA, meaning that we cannot distinguish between switch and backup because both reactions in the coessential pair can indistinctly play both roles. As a consequence, the corresponding internal connected component is only formed by the switch and the backup while the SL cluster contains other reactions. We found no other topologically invariant coessential PSL pairs in the organisms.

### Functional and structural cost of essential plasticity

The fact that coessential partners of switch reactions in PSL pairs remain silent in the WT and only change their activation state when needed to ensure the viability of the organism points to possible costs associated to the activation of these backup systems. To check this hypothesis, we measured both structural and functional costs associated to the viability of PSL mutants. Notice that the biomass yield is not a good indicator of possible functional costs. In more than 80% of the mutants the FBA solution is suboptimal but very close to optimal, while growth is not changed with respect to the WT in the rest.

**Fig 2:**
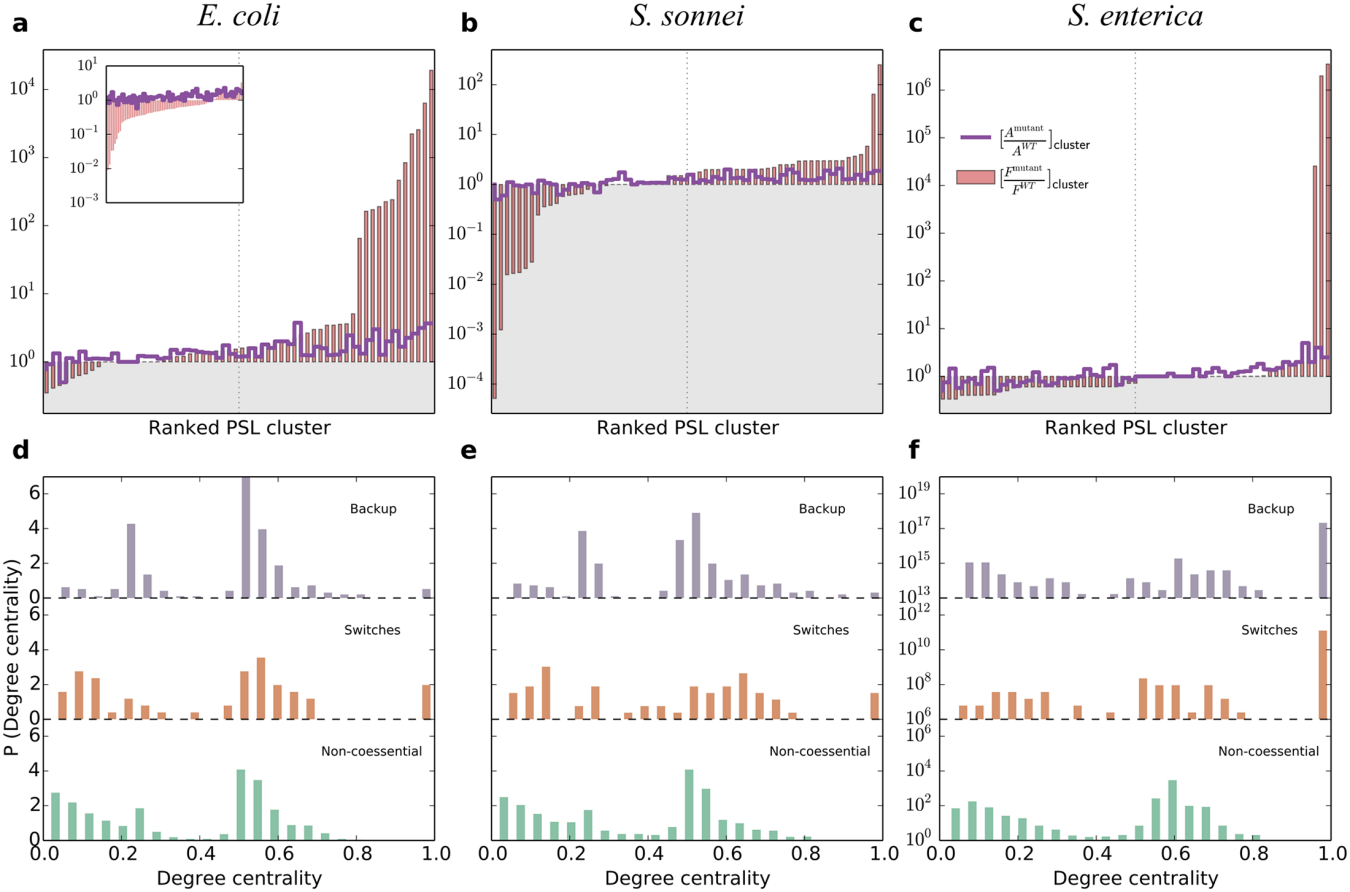
Cost of essential plasticity. **a–c) Functional cost.** For the three bacteria in minimal medium, we show the PSL-mutant–to–WT ratio of the total flux running through SL clusters (bars), the number of active reactions in SL clusters (continuous line), and the ATP production rate obtained by optimizing growth (discontinuous line). The grey shaded area indicates the region where the PSL mutants have values smaller than in the WT. The dotted vertical lines indicate half of the number of PSL mutants. **Inset a.** The same as in **a** for rich medium. **d–f) Structural cost.** Distributions of the degree centrality of reactions belonging to the switch, backup, and noncoessential reactions inside SL clusters for the three organisms considered.

We quantified the flux and energetic requirements of PSL mutants as compared to the WT. The flux disparity measure is given by the ratio of the total flux running through reactions in SL clusters in mutants relative to that of the same reactions in the WT, [*F*^*mutant*^/*F*^*WT*^]_*cluster*_. The results in (Fig 2a–2c) show that this quantity is specific to the mutant. However, in *E. coli* and *S. sonnei* more than 70% of the clusters have a mutant-to-WT flux ratio larger than one with average values 542.7 and 6.6, respectively. Conversely, the flux through more than half of the SL clusters of *S. enterica* is lower in PSL mutants than in the WT, but flux decreases are relatively minor in most PSL mutants while flux increases are dramatic, such that the average of the ratio over mutants is more than 90000 (Table 1).

The observed flux increase is not only related to the larger number of active reactions in SL clusters of mutants as compared to the WT, (Fig 2a–2c) and S1 Fig, but also to a higher average flux per active reaction, see S2 Fig. More specifically, 53 mutants in *E. coli* have more active reactions in their SL cluster than in the WT, and the average flux per active reaction is higher in 69% of the clusters. In *S. sonnei*, the number of mutants with more active reactions in the SL cluster than in the WT is still very high, 49 of 63, but the situation is more balanced regarding the average flux per active reaction. In *S. enterica*, still 40 of the 61 mutants have the same or more active reactions in SL clusters but the average flux per active reaction is generally lower.

The second magnitude calibrating the functional cost of viability in PSL mutants is given by the ratio of ATP production in the mutants as compared to the WT, *E*^*mutant*^/*E*^*WT*^. ATP production is defined as the flux through the ATP maintenance reaction –which is a balanced ATP hydrolysis reaction used to simulate energy demands not associated with growth– versus the intake flux of oxygen, both obtained by optimizing biomass yield [7]. The flux through the ATP maintenance reaction is preserved and deviations of the ATP production in some mutants as compared to WT are basically due to changes in oxygen consumption, see S3 Fig. The number of mutants showing this altered phenotype is 35% in *E. coli* and 41% in *S. sonnei*. Oxygen needs for the production of the same ATP amount in the affected *E. coli* mutants is always increased, while it is always decreased in altered *S. sonnei* mutants. Curiously, the variations in oxygen consumption in mutants of both bacteria is in all cases a fixed amount.

The increased number of active reactions in most mutants gives a rough indication of the cost of essential plasticity at the structural level. A different estimation can be obtained by evaluating the degree centrality [23] of reactions within the internal connected component of a SL cluster, which informs about their role in mediating interactions among other reactions. The investigation of this quantity for the three bacteria, shown in (Fig 2d–2f) (the results are qualitatively similar for other centrality measures), reveals that PSL coessential reactions, and especially backups, have higher centrality as compared to noncoessential reactions. The centrality distribution of switches features a tendency towards lower centrality values, but they always possess a marked peak at very large values. This makes them, on average, more central than noncoessential reactions. Finally, backup reactions have a distribution which is more concentrated at intermediate-to-large centrality values. In fact, the probability that backups have higher centrality than switches and than noncoessential reactions in *E. coli* and *S. sonnei* is approximately 0.64 and 0.55, respectively, while the value for *S. enterica* is notably smaller, 0.37.

### Entanglement of SL clusters by overlapping reactions

SL clusters are not disjoint but strongly coalescent in their component reactions and associated genes. The first observation supporting this is given by the repeated usage of the same backup system for different switches in an organism. This redundancy is intensive in *E. coli* and *S. sonnei*, in which ≈ 20% of the switches share what we call the “fatty acid biosynthesis backup system” formed by eleven reactions (the “fatty acid biosynthesis backup system” includes the four 3-oxoacyl-[acyl-carrier-protein] synthase, the three 3-oxoacyl-[acyl-carrier-protein] reductase reactions, and the four 3-hydroxyacyl-[acyl-carrier-protein] dehydratase reactions in the bacteria). This specific redundancy explains the prominent function of Cell Envelope Biosysthesis as a backup for Membrane Lipid Metabolism, with a concentration of PSL pairs at their interface [7].

**Fig 3:**
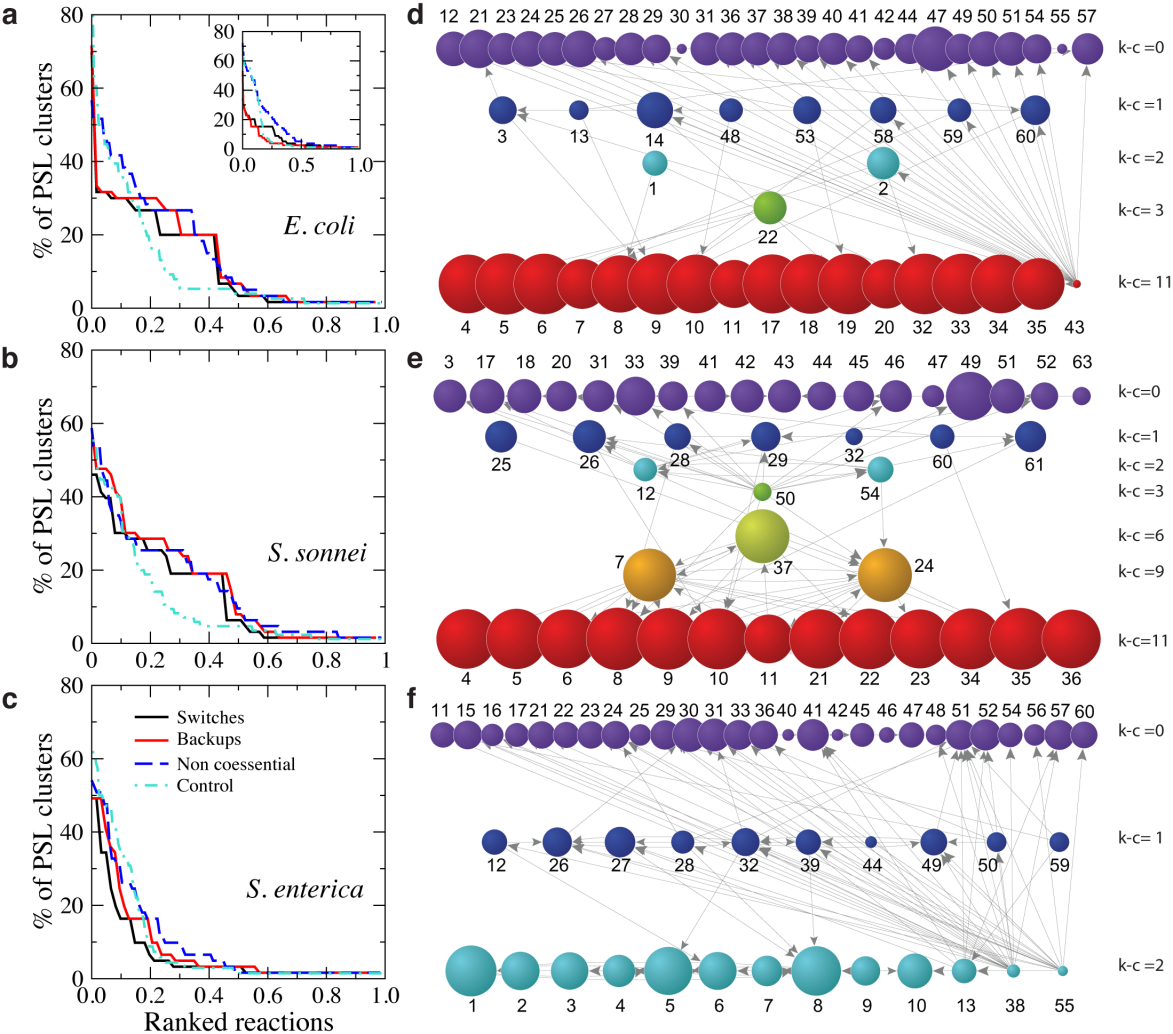
Redundancy of reactions in SL clusters. **a–c**: **Ranking of reactions ordered by number of occurrences in different SL clusters in minimal medium**. Curves for switches, backup, and non–coessential reactions are shown. The control curve corresponds to occurrences of active non single essential or coessential reactions in the SL clusters of the corresponding mutants. **a Inset**. The same as in **a** in rich medium. **d–f**: **Entanglement of SL clusters by overlapping reactions.** Each graph is obtained by considering that two SL clusters are connected by a directed link from SL cluster *i* to SL cluster *j* if at least 75% of the reactions in *i* are also in *j*. Nodes represent SL clusters. The size of a node is proportional to the size of the corresponding SL cluster in number of reactions and the color indicates its coreness index k-c (see Materials and methods). Notice that, in this representation, the connections between nodes in the same k-core may lay beneath the nodes, see S9 Fig for a different display. Each SL cluster is identified by a number, see S1, S2 and S3 Tables for details.

The redundancy not only affects backup reactions. We ranked all reactions participating in SL clusters by their occurrence in different clusters. Results are shown in (Fig 3a–3c). We compared the rankings of switches, backups and noncoessential reactions with the control given by the sets of differentially activated reactions resulting when knocking down reactions which are active in WT, but which are not essential or coessential. As expected, the profiles of participation in SL clusters of switches and backups are similar. In fact, the p-values of the K-S tests (Table 1) do not allow to exclude that switch and backup reactions have the same distribution for all organisms, while these are different from the curves of the control test, which decreases much more quickly in *E. coli* and *S. sonnei* indicating a stronger overlap of SL clusters as compared with rearrangements caused by random deletion of reactions. In line with what has been observed so far, the picture changes in the case of *S. enterica*, with no clear distinction between switches, backup, and control reactions.

As a common trend in the three bacteria, we observe a heterogeneity of participation values such that a small number of reactions participates in a very large fraction of clusters. This suggests that metabolic reorganizations caused by essential plasticity happen mainly by leveraging on some key reactions. For instance, the reductase reactions producing Isopentenyl diphosphate and Dimethylallyl diphosphate, used by organisms in the biosynthesis of terpenes and terpenoids, form a SL cluster in *E. coli* and coappear in 70% of all SL clusters in this organism. The two reactions of this SL cluster are totally exchangeable in terms of assuming the switch or backup role. All the statistics measured to characterize the functional and structural cost of essential plasticity remain invariant when the roles are swapped, and their FVA flux ranges in optimal states are identical. Another intriguing result is related with oxygen consumption in mutants of *E. coli* and *S. sonnei*. Both organisms display a strong overlap of SL clusters of the mutants which display an altered consumption of oxygen in comparison with WT. Strikingly, all of them share a module of three reactions for the exchange, transport and oxidation of iron, which never participate in SL clusters of mutants which do not show alteration of oxygen consumption. This is in agreement with observations that report oxygen as a signal for regulating iron acquisition in Shigella [24].

The strong entanglement of SL clusters can also be explored by constructing the participation backbone. This is a graph representations in which two clusters *i* and *j* are connected by a directed link from *i* to *j* if the proportion of reactions in cluster *i* which are also in cluster *j* is larger than a certain significance level. The unfiltered participation graph in which two clusters are linked whenever they share at least one reaction is almost fully connected and not very informative. In order to give a meaningful backbone, the significance level is chosen to optimize the trade-off between preserving the maximum number of clusters while reducing the number of links to just those which are significant [25], see S4 Fig. For the three bacteria, we have used a significance level of 75%, meaning that two clusters are connected if more than 75% of the reactions in one are also in the other, (Fig 3d–3f). We have performed other complementary tests, see the network representation of pathways entangled through PSL pairs S5 Fig and reaction entanglement matrices showing co-occurrences of reactions in SL clusters, S6, S7 and S8 Figs. The sparsity of the backbones allows us to study quantitatively the hierarchical ordering of SL clusters using the k-core decomposition [26], which identifies groups of clusters having k-c or more connections among them (see Materials and methods). Each SL cluster is annotated with the k-c index given by the maximum k-core it belongs to, computed on the basis of outgoing connections. Notice that SL clusters in the highest k-cores are very densely connected among them. In *E. coli* and *S. sonnei*, the k-core partition denotes a hierarchical core-pheriphery structure. SL clusters are clearly separated into two groups with low and high k-c value, the latter typically formed by the largest SL clusters. The only exception is the size-two SL cluster in *E. coli* Isopentenyl- Dimethylallyl diphosphate mentioned above, which acts as an important interface between the core and many peripheral clusters, see (Fig 3d) and S9 Fig. The core contains approximately 1/5 of the SL clusters and forms an almost fully connected set of reactions participating in many other SL clusters. In contrast, the k-core layout of *S. enterica* is almost flat with no relevant core-periphery structure.

### Entanglement of SL clusters by overlapping genes

**Fig 4:**
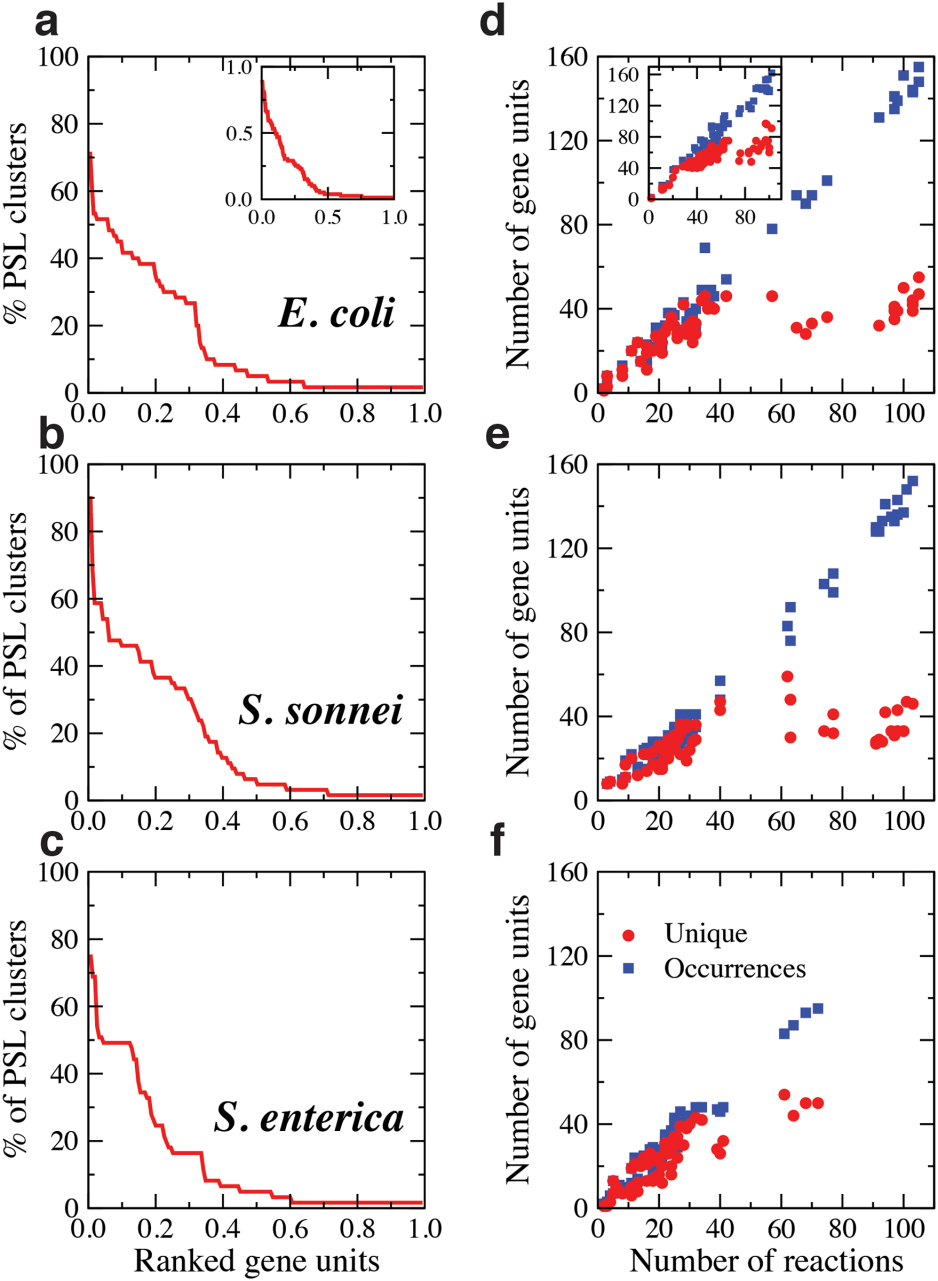
Redundancy of genes in SL clusters. a-c. Metabolic gene units (gene and gene complexes) ranked according to the number of SL clusters in which they participate. **d-f** Number of gene units *vs* number of reactions in SL clusters. For each organism considered, number of gene units associated to reactions in each SL cluster versus the number of corresponding reactions, including gene repetitions (labeled Occurrences) and excluding them (labeled Unique).

At the level of genes, the entanglement of SL clusters is even stronger. Here, we consider genetic units, which can be single genes or gene complexes (sets of functionally related genes that regulate together a metabolic reaction in a SL cluster via an AND logical relation). To clarify the question whether there is a common set of regulatory genes for SL clusters we ranked metabolic genes according to the number of SL clusters in which they participate, results in (Fig 4a–4c). Hub genes participate in more than 70% of SL clusters and the top 10% enter in about 50% of the sets. One example is the gene regulating the function of the reductase reactions Isopentenyl and Dimethylallyl diphosphate in *E. coli*. This gene appears to be involved in cell lysis and in the stress response of bacteria in reaction to amino-acid starvation, fatty acid limitation, iron limitation, heat shock and other stress conditions. Its action causes the cell to divert resources away from growth and division toward amino acid synthesis in order to promote survival until nutrient conditions improve. However, more than 50% of the genes is specific to up to three SL clusters. Interestingly, the gene participation curve decays faster for *S. enterica* than for the other two bacteria, highlighting again a more limited organization.

In (Fig 4d–4f), we plot both the number of unique genes entering in a cluster (Unique) and their total number by counting repetitions (Occurrences). The number of occurrences grows linearly with the number of reactions in each set, with an approximate slope of 1.4 in the three bacteria, which indicates that complexes are frequently associated to the regulation of SL clusters. Interestingly, the number of unique genes follows the same linear growth up to a ‘critical’ value from which it saturates to a constant around 40 for SL clusters with more than ∼60 reactions (around half the maximum size of SL clusters). The saturation effect implies that large SL clusters are regulated by a reduced number of different genes and that the basis of regulation of genes and the role of complexes grows with the number of reactions in the set. These features are common to the three organisms, although SL clusters in *S. enterica* are smaller than in the other two bacteria, so that the saturation effect is less evident and the redundancy of genes is more limited. Pairs of genetic units show also a clear tendency to co-occurrence in SL clusters, see the gene entangle matrices for the three bacteria in S10, S11 and S12 Figs.

### Sensitivity to different optimal states and environmental conditions in *E. coli*

To check the dependency of SL clusters on alternative optimal FBA solutions in glucose minimal medium, we have analyzed the congruency of the detected SL clusters in *E. coli* with the range of possible reaction fluxes, as determined by FVA [27] fixing the optimal growth rate (see Materials and methods). We found that 68% of the clusters, 41 out of 60, present optimal flux ranges which are fully consistent with the solution reported in the previous sections, meaning that the minimum flux attainable by switch reactions ensures that they are active while, simultaneously, the flux of the corresponding backup reactions is basically confined to be zero in optimal states. In 5 more cases the maximum allowed flux through the switch reaction is at least a hundred times larger than that of the corresponding backups. Only 4 clusters, less than 7%, show backup systems with all reactions practically bounded to zero except for one of them, which presents a potential maximum flux comparable to that of the switch reaction. However, the biomass yield is slightly decreased in the corresponding mutant as compared to the WT, as a consequence of the activation in the backup system of reactions which are bounded to zero flux in the optimal states. Finally, the remaining 10 SL clusters correspond to alternative optimal solutions in which the switch and the backup system can exchange role in terms of optimal biomass production, although flux and energetic requirements of the mutants can be different.

Results were also obtained for *E. coli* in rich medium (see Materials and methods). The total number of different reactions in SL clusters is a factor 1.8 larger than in minimal medium and the average number of backups per switch increases approximately in the same proportion. The number of switches is slightly increased (Table 1). Similar to glucose minimal medium, the size distribution of SL clusters has a longer tail as compared with the control, inset (Fig 1b), and SL clusters of differentially activated reactions are formed basically by a connected component, inset (Fig 1e). Of the 287 reactions in SL clusters in glucose minimal medium, 85%(244) also belong to SL clusters in rich medium. Of them, only 8.6%(21) change role. In particular, 15 which were coessential are rescued and become nonessential in rich medium. More than 70% of switches and backups in glucose minimal medium are conserved, 43 and 42 respectively. Of them, 40 switches preserve exactly the same backup system, 2 switches acquire an extra backup reaction, and 1, corresponding to the switch reaction isopentenyl pyrophosphate isomerization, changes backup. In this case, the isomers Isopentenyl diphosphate, less reactive, and Dimethylallyl diphosphate, more reactive, can only be produced by the corresponding reductase reaction and by the isopentenyl pyrophosphate isomerization reaction. In glucose minimal medium, the more reactive form is obtained via the less reactive isomerase and not directly by the action of the corresponding reductase, so that the reductase producing Isopentenyl diphosphate and the isomerization reaction act as switches with the reductase reaction producing directly Dimethylallyl diphosphate as a backup. The reverse is observed in rich medium, where the less reactive form is obtained from the more reactive one so that the reductase producing Dimethylallyl diphosphate and the isomerization reaction act as switches with the reductase reaction producing directly Isopentenyl diphosphate as a backup. Interestingly, in two more SL clusters the switch and the backup swap roles so that alternative mechanisms are used to produce specific metabolites. One of them is related to the production of deoxyuridylic acid, an intermediate in the metabolism of deoxyribonucleotides. The hidrolase reaction, without the direct intervention of ATP, is active in glucose minimal medium while the active reaction in rich medium is the thymidine kinase catalysed reaction, which involves the direct consumption of ATP.

We see that the cost and energetic requirements of rearrangements from WT to mutant do vary between minimal and rich medium conditions (Fig 2). The flux ratio per module *F*^mutant^/*F*^WT^ tends in fact to generally decrease, at odds with the minimal medium case. This is however reasonable, since in the rich medium there are several metabolic routes that connect nutrients to biomass. Introducing mutations may disrupt many of these routes (literally switching off the metabolism of some of the redundant nutrients) without impairing survival, decreasing the total flux running through the modules as an effect. In the minimal medium case, instead, it is impossible to disrupt these routes without incurring in lethality. Besides this discrepancy, switches and backup participations in different SL clusters decrease faster than non coessential and control reactions, suggesting that SL cluster entanglement decreases dramatically in the presence of multiple nutrients, as also suggested by the reduced average number of backups per switch.

## Discussion

Metabolic networks can change their state significantly without causing the loss of an organism’s ability to survive in a given environment, and this property allows it to explore a wide range of novel metabolic abilities [28]. However, flux rerouting has been claimed as negligible in providing robustness for a large number of mutant strains [29–31]. Nevertheless, metabolic flux reorganization becomes essential in some critical situations, in particular when reactions in synthetic lethal pairs fail. The big majority of SL interactions in the studied bacteria involve the activation of silent backup coessential partners to ensure the viability of the mutants. This essential plasticity is mediated by the activation and inactivation of reactions in SL clusters acting as backup systems containing coessential but also nonessential or coessential reactions, in contrast to the model of SL interactions restricted to coessential pairs.

The robustness that confers essential plasticity comes at the expenses of acquired vulnerabilities and of increased structural and functional costs. These costs are manifested in an increased number of active reactions and total flux running through SL clusters, lower efficiency in energetic production, a slightly reduced biomass yield, and an increased centrality of reactions in the backup systems. We hypothesize that, despite the increased functional and structural cost of viability in SL mutants, the expected burden for sustaining alternative backup systems for all switch reactions in the organism is indeed buffered by the overlap of SL clusters. This is supported by our observation that SL cluster entanglement decreases markedly in the presence of multiple nutrients. The regulation of small sets of enzyme-coding genes controlling a reduced number of differentially activated reactions which participate in many different SL clusters is sufficient to provide the varied combinations necessary to protect the bacterium against a diversity of switch mutations. We believe that the higher centrality of backups, which act as a sort of local hubs in SL clusters, is related to the requirement of an efficient regulation of metabolism –e.g., by transcriptional regulation [32]– controlling the alternative metabolic flux routing within the network in order to sustain viability in the event of a harmful mutation, like the knockout of a switch. This comes at the expenses of an increased vulnerability of the organisms in the new mutated state, since the increased centrality of backup reactions as compared to switches makes the metabolic structure of the mutant more fragile and vulnerable to potential failures [33], and suggests a tradeoff between robustness and the efficiency of backup regulation.

The existence of SL clusters and their strong entanglement in minimal medium is a common feature in the three bacteria analysed here. The entanglement of SL clusters is observed at two different levels. First, silent PSL coessential reactions tend to form leagued backup systems which are shared by several switches. Second, clusters are also entangled by other noncoessential reactions. However, some specific features seem more sensitive to evolutionary pressure, as revealed by a comparative analysis of the results for the three species. In fact, *E. coli* and *S. sonnei* are extremely congruent, in accordance with the very short phylogenetic distance separating them in the evolutionary tree and with previous results claiming members of the genus Shigella as “*Eschericia coli* in disguise” [16, 17]. Differences are however interesting, like the patterns of oxygen consumption observed in a high proportion of SL mutants versus WT. The increase in *E. coli* implies a reduced efficiency in energy production by oxidative processes. The decrease in *S. sonnei* denotes a change of strategy in the production of energy from aerobic to anaerobic mechanisms. On the other hand, *S. enterica* shows a differentiated profile, less complex and structured, in line with its role as evolutionary ancestor. A signature of the limited complexity of *S. enterica* is the fact that, in contrast to the case of *E. coli* and *S. sonnei*, no clear distinction was observed in terms of the occurrence of switches, backups, and control reactions in its SL clusters, (Fig 3a–3c). A large set of switches and backups in this bacterium shows a decreased participation in SL clusters as compared to the other two bacteria. This means that the entanglement of SL clusters is weaker in *S. enterica*, which implies that essential plasticity is less efficient in reducing the costs associated to the maintenance of backup systems in this organism. Overall, the results obtained in minimal medium suggest that *E. coli* and *S. sonnei* are more optimized towards growth and biomass yield and undergo a much more complex metabolic reorganization to face mutations and still achieve performances comparable to the WT.

Essential plasticity, a more sophisticated mechanism as compared to redundant metabolic flows in other SL interactions, seems to be promoted by evolutionary pressure but, at the same time, tends to increase the degeneracy and centrality of backup systems as a regulatory mechanism ensuring the entanglement of SL clusters forming a hierarchical core-periphery structure. This entanglement economizes the huge potential metabolic burden due to the maintenance alternative metabolic routes for all PSL interactions in an organism. On the other hand, the flux reorganization of *E. coli* in rich medium, in which cluster entanglement decreases markedly, lead us to think that the strength of entanglement can be responsive to environmental stresses, like starvation.

### Conclusion

In summary, we propose a change of paradigm in the approach to understand the phenomenon of synthetic lethality. The complexity of molecular interactions at the cell level urge us to go from the mere screening of SL reaction or gene pairs, or even of triplets or higher order motifs, to the study of SL clusters and their entanglement. Approaching directly SL pairs of reactions or genes without their multifunctional integration in clusters is like drawing paths between pairs of geographical places without the scaffold of a map telling how the different paths relate to each other. The complete portray at the systems level is far more complex than a collection of separate PSL pairs. Beyond theoretical implications for the understanding of plasticity in metabolic networks, our results could help to identify drug action and to design improved strategies that reduce undesired resistance in synthetic lethal interactions to chemicals in pathogens. We believe that SL clusters will be also found in human cells, with important implications for biomedicine and biotechnology. Our work reveals that backups that belong to the same SL cluster offer alternative but equivalent targets, a clear advantage in cases in which the experimental targeting of some specific reaction is technically more difficult. At the same time, not all computationally detected SL pairs have the same quality as potential therapeutic targets in complex diseases such as cancer or to fight infections of pathogens. We expect that more redundant coessential reactions with a higher participation in different SL clusters can become efficient and reliable supertargets.

## Materials and methods

### Statistics of the genome-scale metabolic networks

In Table 1, and for each of the three bacteria, we report the metabolic reconstruction (Organism, Model, Reference), the number of reactions included in the genome-scale reconstruction (*N*_*R*_ all), the number of reactions possibly active according to FVA (*N*_*R*_ FVA), the number of active reactions in the FBA solution in glucose minimal medium (*N*_*R*_ active), the number of metabolites in the reconstruction (*N*_*M*_ actual) and those resulting when considering compartments (*N*_*M*_ synth), the number of single essential reactions, the number of Plasticity SL pairs, and of Redundancy SL pairs, the number of SL clusters and switches (*R*_*switches*_), and the number of backups (*R*_*backups*_), noncoessential reactions (*R*_*noncoess*_), total different reactions (Total *R*), and Gene units associated to the SL clusters. We also report the ratio of the total flux running through reactions in SL clusters of mutants and WT ([*F*_*mutant*_/*F*_*WT*_]_*cluster*_), the ratio of active reactions in SL clusters of mutants and in the WT (*A*_*NG*,*mutant*_/*A*_*WT*_), and the ratio of ATP production in PSL mutants and in the WT (*E*_*NG*,*mutant*_/*E*_*NG*,*WT*_). In relation to centrality measures, we report the fraction of times that backups have higher centrality than switches inside the internal connected component of SL clusters *C*_*BgtS*_, the fraction of times that backups have higher centrality than non coessential reactions inside the internal connected component of SL clusters *C*_*BgtNC*_, the average centrality of backup reactions in the internal connected component of SL clusters 〈*C*_*B*_〉, the average centrality of switch reactions in the internal connected component of SL clusters 〈*C*_*S*_〉, the average centrality of noncoessential reactions in the internal connected component of SL clusters 〈*C*_*N*_*CE*〉. For *E. coli*, values in parentheses correspond to rich medium, see next subsection. We have also computed the p-values of the Kolmogorov-Smirnov (K-S) test for the different distributions that we obtain in our analysis. More specifically, we have calculated the distances between the distributions of observed SL cluster sizes and the distributions given by the control shown in (Fig 1b–1d) (p-values in row *SL cluster sizes*), and between the curves associated to the rankings of occurrences of reactions in different SL clusters as shown in (Fig 3a–3c) (p-values in rows *Ranking in clusters*, where S stands for Switch, B for Backups, and NC for Noncoessential. The rest of potential comparison, for instance between S or B with the control, have p-values which are virtually zero and are not included in the table).

### Flux balance analysis, flux variability analysis, and environmental conditions

Flux Balance Analysis (FBA) [15] is a technique which allows to compute metabolic fluxes without the need of kinetic parameters, just by using constrained-optimization. The vector of the time variation of the concentrations of metabolites $\dot{c}$ is related with the stoichiometric matrix *S* of the whole network (it contains the stoichiometric coefficients of each metabolite in each reaction of the network) and the vector of fluxes $\nu ,\dot{c}=S\cdot \nu$. Steady-state is assumed, thus *S* ⋅ *ν* = 0. In general, metabolic networks contain more reactions than metabolites, and hence the system of equations for the fluxes is underdetermined. Hence, a biological objective function must be defined in order to select a biologically meaningful solution. In this work, we use FBA to find the solution that optimizes the growth of the organism, which is equivalent to maximize biomass formation. Reversibility of reactions is also added in order to constrain the solutions. Since we have a linear system of equations with linear constraints, Linear Programming is used in order to compute a flux solution in a small amount of time (of the order of 1 s), which implies a computationally cheap method.

Flux variability analysis [27] is computed by fixing the biomass yield to its optimal value and by extracting the maximum and minimum flux value associated to each reaction in this fixed optimal condition via linear programming.

According to the specifications in each metabolic reconstruction, growth in glucose minimal medium was simulated by fixing the lower bound of the glucose exchange reaction to −10*mmol*/(*gDW* ⋅ *h*) for *E. coli* and *S. sonnei*, and to −5*mmol*/(*gDW* ⋅ *h*) for *S. enterica*.

For the rich medium in *E. coli*, we used a Luria-Bertani Broth [34], which contains as additional compounds purines and pirimidines apart from amino acids. We also added vitamins, namely biotin, pyridoxine, and thiamin, and also the nucleotide nicotinamide monocleotide [31]. Other compounds, like PABA or chorismate, cannot be uptaken by the *E. coli* model that we are using. The exchange constraints bounds of these compounds are set to −10*mmol*/(*gDW* ⋅ *h*) $\left(\nu_{exchange}^{compound}\geq -10\right)$. A detailed list of the added compounds is given in S4 Table.

### Detection of SL pairs

From the set of reactions in the genome-scale reconstructions, we excluded essential reactions detected computationally and also spontaneous reactions (e.g transport reactions). We focused exclusively on reactions catalyzed by enzymes with an associated gene. In this way, we identified a set of candidate reactions in each organism that can be removed individually, but whose pair deletion may be lethal for the organism. We checked every possible pair by applying FBA to the double mutant. As in [7], we classified the detected SL pairs into plastic and redundant, depending on whether only one or both reactions are active in the FBA solution in the given medium.

### Computation of the centrality of reactions in SL clusters

We modelled metabolism as a bipartite directed network [35], where directed links connect metabolites with reactions in which they participate as reactants or products. The degree centrality of a reaction is simply given by its degree *k*, measuring the number of other reactions connected to it by shared metabolites. We define the normalized degree centrality of a reaction in the internal connected component of a SL cluster as *k*_*r*_/(*R* − 1), where *k*_*r*_ stands for the number of reactions connected to reaction *r* inside the connected component, and *R* is its total number of reactions.

### Definition of k-core in complex directed networks

In complex networks, the k-core decomposition of a graph allows to disentangle the hierarchical structure of networks by progressively focusing on their central cores. It is a threshold-based hierarchical decomposition of a graph. A k-core of level *c* is defined as the maximal subgraph in which all the nodes have at least *c* internal connections [26]. It can be obtained by recursively deleting all nodes with less than *c* connections until all nodes in the remaining graph have at least *c* neighbors. Notice that the obtained cores for different values of *c* form a nested hierarchy of subgraphs. As a consequence, each node in a network can be labeled with an index *k* − *c* called its coreness, meaning that the node belongs to the *k* − *c* core but has been removed by the process in the *k* − *c* + 1 core.

In the case of directed networks, in which nodes have both incoming and outgoing neighbours, the k-core decomposition can be based on the incoming connections, the outgoing connections, or a combination of the two. In this work, we based our definition on outgoing connections, such that the coreness of a node, which represents a SL cluster, is given by its maximum k-core out, where a k-core out with value *k* − *c* is defined as the maximal subgraph of SL clusters such that all the SL clusters in it have at least *k* − *c* outgoing connections inside the subgraph.

## Supporting information

S1 Table Data analyzed in the paper for the organism *E. coli* in minimal medium growth condition. The table reports FBA solution, PSL Clusters, control clusters, gene clusters and cluster entanglement for the organism *E. coli* in minimal medium.
(XLSX)

S2 Table Data analyzed in the paper for the organism *S. sonnei* in minimal medium growth condition. The table reports FBA solution, PSL Clusters, control clusters, gene clusters and cluster entanglement for the organism *S. sonnei* in minimal medium.
(XLSX)

S3 Table Data analyzed in the paper for the organism *S. enterica* in minimal medium growth condition. The table reports FBA solution, PSL Clusters, control clusters, gene clusters and cluster entanglement for the organism *S. enterica* in minimal medium.
(XLSX)

S4 Table Data analyzed in the paper for the organism *E. coli* in rich medium growth condition. The table reports FBA solution, PSL Clusters, control clusters, gene clusters and cluster entanglement for the organism *E. coli* in rich medium.
(XLSX)

S1 Fig Number of active reactions in PSL clusters of mutants versus WT for the three bacteria. Scatter plots in which each dot represents a PSL cluster and the diagonal line denotes equal number of active reactions.
(TIF)

S2 Fig Average flux per active reaction in PSL clusters of mutants versus WT for the three bacteria. Scatter plots in which each dot represents a PSL cluster and the diagonal line denotes equal average fluxes.
(TIF)

S3 Fig ATP production and oxygen consumption of mutants versus WT for the three bacteria. Each dot represents a PSL cluster.
(TIF)

S4 Fig Statistics of links and nodes in the backbones of the three bacteria as a function of the overlap threshold. In a backbone, two PSL clusters are connected by a directed link from PSL cluster *i* to PSL cluster *j* if more than a certain fraction of reactions in *i*, given by the overlap threshold, are also in *j*. Left column: number of clusters and links remaining in the backbone as a function of the threshold. Right columns: fraction of clusters as a function of the fraction of links remaining in the backbone as the threshold is varied. Dashed lines mark the threshold used in the main text and in the next figure. Notice that, for the three bacteria, the region around 75% for the overlap threshold gives a good compromise between preserving the maximum number of clusters in the backbone and reducing the number of links to the minimum.
(TIF)

S5 Fig Back-up cell envelope-membrane lipid for the three types of bacteria. We annotated reactions in PSL pairs in terms of biochemical pathways to provide a network representation where pathways are nodes linked whenever they participate together in a PSL interaction. The network summarizes which pathways are, overall, a metabolic backup of others, for the case of **(a)** *E. coli*, **(b)** *S. sonnei* and **(c)** *S. enterica*. We observe that PSL pairs happen mostly intra-pathway, with the striking exception of the strong entanglement between Cell Envelope Biosysthesis and Membrane Lipid Metabolism in *E. coli* and *S. sonnei*.
(TIF)

S6 Fig Entanglement matrix for pairs of reactions in PSL clusters of *E. coli*. Each matrix shows the number of clusters in which a pair of reactions in PSL clusters coappear. Reactions are ordered according to their metabolic pathway. Pathways are numbered and reported in the list below the matrices. The type of reactions (switch, backup, noncoessential) in the pair is denoted by the color key besides the matrices, e.g. switch reactions are in red. Each entry in the matrix corresponds to the number of PSL clusters in which the corresponding pair of reactions coappear.
(TIF)

S7 Fig Entanglement matrix for pairs of reactions in PSL clusters of *S. sonnei*. Each matrix shows the number of clusters in which a pair of reactions in PSL clusters coappear. Reactions are ordered according to their metabolic pathway. Pathways are numbered and reported in the list below the matrices. The type of reactions (switch, backup, noncoessential) in the pair is denoted by the color key besides the matrices, e.g. switch reactions are in red. Each entry in the matrix corresponds to the number of PSL clusters in which the corresponding pair of reactions coappear.
(TIF)

S8 Fig Entanglement matrix for pairs of reactions in PSL clusters of *S. enterica*. Each matrix shows the number of clusters in which a pair of reactions in PSL clusters coappear. Reactions are ordered according to their metabolic pathway. Pathways are numbered and reported in the list below the matrices. The type of reactions (switch, backup, noncoessential) in the pair is denoted by the color key besides the matrices, e.g. switch reactions are in red. Each entry in the matrix corresponds to the number of PSL clusters in which the corresponding pair of reactions coappear.
(TIF)

S9 Fig Entanglement of PSL clusters by overlapping reactions in *E. coli*, minimal medium. The graph is obtained by considering that two PSL clusters are connected by a directed link from PSL cluster *i* to PSL cluster *j* if at least 75% of the reactions in *i* are also in *j*. Nodes represent PSL clusters. The size of a node is proportional to the size of the corresponding PSL cluster in number of reactions and the color indicates its maximum k-core out, where a k-core out *k* − *c* is defined as the maximal subgraph of PSL clusters such that all the PSL clusters in it have at least *k* − *c* outgoing connections inside the subgraph.
(TIFF)

S10 Fig Entanglement matrix for pairs of genes or gene complexes in PSL clusters of *E. coli*. Each matrix shows the number of sets in which a pair of genetic units (gene or gene complexes) in PSL clusters coappear. Each complex is composed of a number of genes varying from 1 up to 13 and may appear more than once in each set. For this reason, pairs of gene complexes may have a cooccurrence frequency that exceeds the number of sets, as it can be observed mostly in the upper diagonal part of the matrices. The number of genes in the complex is denoted by the color key beside the matrix (e.g. red denotes single genes).
(TIF)

S11 Fig Entanglement matrix for pairs of genes or gene complexes in PSL clusters of *S. sonnei*. Each matrix shows the number of sets in which a pair of genetic units (gene or gene complexes) in PSL clusters coappear. Each complex is composed of a number of genes varying from 1 up to 13 and may appear more than once in each set. For this reason, pairs of gene complexes may have a cooccurrence frequency that exceeds the number of sets, as it can be observed mostly in the upper diagonal part of the matrices. The number of genes in the complex is denoted by the color key beside the matrix (e.g. red denotes single genes).
(TIF)

S12 Fig Entanglement matrix for pairs of genes or gene complexes in PSL clusters of *S. enterica*. Each matrix shows the number of sets in which a pair of genetic units (gene or gene complexes) in PSL clusters coappear. Each complex is composed of a number of genes varying from 1 up to 13 and may appear more than once in each set. For this reason, pairs of gene complexes may have a cooccurrence frequency that exceeds the number of sets, as it can be observed mostly in the upper diagonal part of the matrices. The number of genes in the complex is denoted by the color key beside the matrix (e.g. red denotes single genes).
(TIF)

## References

[1] Wagner A. Robustness and evolvability in living systems. Princeton, New Jersey, USA: Princeton University Press; 2005.

[2] Baba T, Ara T, Hasegawa M, Takai Y, Okumura Y, Baba M, et al Construction of Escherichia coli K-12 in-frame, single-gene knockout mutants: the Keio collection. Molecular Systems Biology. 2006;2:2006.0008. doi: 10.1038/msb4100050 10.1038/msb4100050 PMC1681482 16738554

[3] Hartman JL, Garvik B, Hartwell L. Principles for the buffering of genetic variation. Science. 2001;291:1001–1004. 11232561 10.1126/science.1056072

[4] Tucker CL, Fields S. Lethal combinations. Nat Genet. 2003;35:204–205. doi: 10.1038/ng1103-204 14593402 10.1038/ng1103-204

[5] Deutscher D, Meilijson I, Kupiec M, Ruppin E. Multiple knockout analysis of genetic robustness in the yeast metabolic network. Nat Genet. 2006;38:993–998. doi: 10.1038/ng1856 16941010 10.1038/ng1856

[6] Suthers PF, Zomorrodi A, Maranas CD. Genome-scale gene/reaction essentiality and synthetic lethality analysis. Mol Syst Biol. 2009;5:301 doi: 10.1038/msb.2009.56 19690570 10.1038/msb.2009.56 PMC2736653

[7] Güell O, Sagués F, Serrano MÁ. Essential plasticity and redundancy of metabolism unveiled by synthetic lethality analysis. PLoS Comput Bio. 2014;10(5):e1003637 doi: 10.1371/journal.pcbi.1003637 24854166 10.1371/journal.pcbi.1003637 PMC4031049

[8] Roemer T, Boone C. Systems-level antimicrobial drug and drug synergy discovery. Nature Chemical Biology. 2013;9:222–231. doi: 10.1038/nchembio.1205 23508188 10.1038/nchembio.1205

[9] Jerby-Arnon L, Pfetzer N, Waldman YY, McGarry L, James D, Shanks E, et al Predicting Cancer-specific Vulnerability via Data-Driven Detection of Synthetic Lethality. Cell. 2014;158:1199–1209. doi: 10.1016/j.cell.2014.07.027 25171417 10.1016/j.cell.2014.07.027

[10] Jeong H, Tombor B, Albert R, Oltvai ZN, Barabási AL. The large-scale organization of metabolic networks. Nature. 2000;407:651–654. doi: 10.1038/35036627 11034217 10.1038/35036627

[11] Ma HW, Zeng AP. The connectivity stucture, giant strong component and centrality of metabolic networks. Bioinformatics. 2003;19:1423–1430. doi: 10.1093/bioinformatics/btg177 12874056 10.1093/bioinformatics/btg177

[12] Guimerà R, Amaral LAN. Functional cartography of complex metabolic networks. Nature. 2005;433:895–900. doi: 10.1038/nature03288 15729348 10.1038/nature03288 PMC2175124

[13] Anderson DI, Levin BR. The biological cost of antibiotic resistance. Curr Opin Microbiol. 1999;2:489–493. doi: 10.1016/S1369-5274(99)00005-3 10508723 10.1016/s1369-5274(99)00005-3

[14] Olivares J, Álvarez-Ortega C, Martinez JL. Metabolic Compensation of Fitness Costs Associated with Overexpression of the Multidrug Efflux Pump MexEF-OprN in *Pseudomonas aeruginosa*. Antimicrobial Agents and Chemotherapy. 2014;58:3904–3913. doi: 10.1128/AAC.00121-14 24777101 10.1128/AAC.00121-14 PMC4068598

[15] Orth JD, Thiele I, Palsson BO. What is Flux Balance Analysis? Nat Biotechnol. 2010;28:245–248. doi: 10.1038/nbt.1614 20212490 10.1038/nbt.1614 PMC3108565

[16] Lan R, Reeves PR. Escherichia coli in disguise: molecular origins of Shigella. Microbes and Infection. 2002;4:1125–1132. doi: 10.1016/S1286-4579(02)01637-4 12361912 10.1016/s1286-4579(02)01637-4

[17] Zuo G, Xu Z, Hao B. *Shigella Strains* Are Not Clones of *Escherichia coli* but Sister Species in the Genus *Escherichia*. Genomics Proteomics Bioinformatics. 2013;11:61–65. doi: 10.1016/j.gpb.2012.11.002 23395177 10.1016/j.gpb.2012.11.002 PMC4357666

[18] Winfield MD, Groisman EA. Phenotypic differences between *Salmonella* and *Escherichia coli* resulting from the disparate regulation of homologous genes. Proceedings of the National Academy of Sciences USA. 2004;101:17162–17167. doi: 10.1073/pnas.0406038101 10.1073/pnas.0406038101 PMC534605 15569938

[19] Orth JD, Conrad TM, Na J, Lerman JA, Nam H, Feist AM, et al A comprehensive genome-scale reconstruction of *Escherichia coli* metabolism—2011. Mol Syst Biol. 2011;7:535 doi: 10.1038/msb.2011.65 21988831 10.1038/msb.2011.65 PMC3261703

[20] Monk JM, Charusanti P, Aziz RK, Lerman JA, Premyodhin N, Orth JD, et al Genome-scale metabolic reconstructions of multiple *Escherichia coli* strains highlight strain-specific adaptations to nutritional environments. Proc Natl Acad Sci USA. 2013;110:20338–20343. doi: 10.1073/pnas.1307797110 24277855 10.1073/pnas.1307797110 PMC3864276

[21] Thiele I, Hyduke DR, Steeb B, Fankam G, Allen DK, Bazzani S, et al A community effort towards a knowledge-base and mathematical model of the human pathogen Salmonella Typhimurium LT2. BMC Systems Biology. 2011;5(1):8 doi: 10.1186/1752-0509-5-8 21244678 10.1186/1752-0509-5-8 PMC3032673

[22] Orth JD, Thiele I, Palsson BO. What is flux balance analysis? Nat Biotechnol. 2010;28:245–248. doi: 10.1038/nbt.1614 20212490 10.1038/nbt.1614 PMC3108565

[23] Newman M. Networks: An Introduction. New York, NY, USA: Oxford University Press, Inc.; 2010.

[24] Boulette ML, Payne SM. Anaerobic Regulation of Shigella flexneri Virulence: ArcA Regulates fur and Iron Acquisition Genes. Journal of Bacteriology. 2007;189:6957–6967. doi: 10.1128/JB.00621-07 17660284 10.1128/JB.00621-07 PMC2045222

[25] Serrano MÁ, Boguñá M, Vespignani A. Extracting the mutiscale backbone of complex weighted networks. Proc Natl Acad Sci USA. 2009;106:6483–6488. doi: 10.1073/pnas.0808904106 19357301 10.1073/pnas.0808904106 PMC2672499

[26] Dorogovtsev SN, Goltsev AV, Mendes JFF. k. Phys Rev Lett. 2006;96:040601 doi: 10.1103/PhysRevLett.96.040601 16486798 10.1103/PhysRevLett.96.040601

[27] Gudmundsson S, Thiele I. Computationally efficient flux variability analysis. BMC Bioinformatics. 2010;11(1):489 doi: 10.1186/1471-2105-11-489 20920235 10.1186/1471-2105-11-489 PMC2963619

[28] Rodrigues JFM, Wagner A. Evolutionary Plasticity and Innovations in Complex Metabolic Reaction Networks. PLoS Comput Biol. 2009;5:e1000613 doi: 10.1371/journal.pcbi.1000613 20019795 10.1371/journal.pcbi.1000613 PMC2785887

[29] Papp B, Pál C, Hurst LD. Metabolic network analysis ofthe causes and evolution of enzyme dispensability in yeast. Nature. 2004;429:661–664. doi: 10.1038/nature02636 15190353 10.1038/nature02636

[30] Blank LM, Kuepfer L, Sauer U. Large-scale ^13^C-flux analysis reveals mechanistic principles of metabolic network robustness to null mutations in yeast. Genome Biol. 2005;6:R49 doi: 10.1186/gb-2005-6-6-r49 15960801 10.1186/gb-2005-6-6-r49 PMC1175969

[31] Wunderlich Z, Mirny LA. Using the topology of metabolic networks to predict viability of mutant straints. Biophys J. 2006;91:2304–2311. doi: 10.1529/biophysj.105.080572 16782788 10.1529/biophysj.105.080572 PMC1557581

[32] DeRisi JL, Iyer VR, Brown PO. Exploring the metabolic and genetic control of gene expression on a genomic scale. Science. 1997;278:680–686. doi: 10.1126/science.278.5338.680 9381177 10.1126/science.278.5338.680

[33] Jeong H, Mason SP, Barabási AL, Oltvai ZN. Lethality and centrality in protein networks. Nature. 2001;411:41–41. doi: 10.1038/35075138 11333967 10.1038/35075138

[34] Sezonov G, Joseleau-Petit D, D’Ari R. *Escherichia* coli physiology in Luria-Bertani broth. J Bacteriol. 2007;189(23):8746–8749. doi: 10.1128/JB.01368-07 17905994 10.1128/JB.01368-07 PMC2168924

[35] Güell O, Sagués F, Serrano MA. Predicting effects of structural stress in a genome-reduced model bacterial metabolism. Sci Rep. 2012;2:621 doi: 10.1038/srep00621 22934134 10.1038/srep00621 PMC3429883

