# Supplementary figures and images for: Metabolic plasticity in synthetic lethal mutants: Viability at higher cost
Source: PLoS Comput Biol. 2018 Jan 30;14(1):e1005949. doi: 10.1371/journal.pcbi.1005949 (PMC5806928; doi:10.1371/journal.pcbi.1005949)

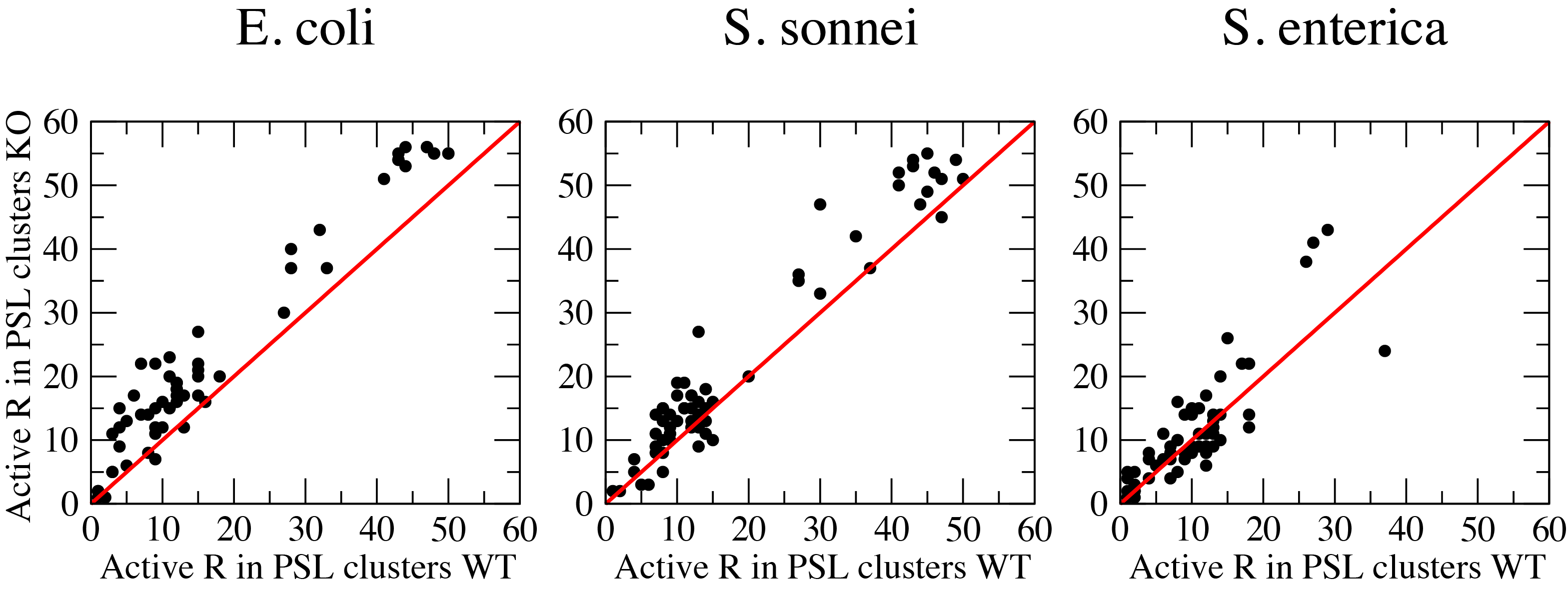

Supplement: S1 Fig — Scatter plots in which each dot represents a PSL cluster and the diagonal line denotes equal number of active reactions. (TIF) [file pcbi.1005949.s005.tif]

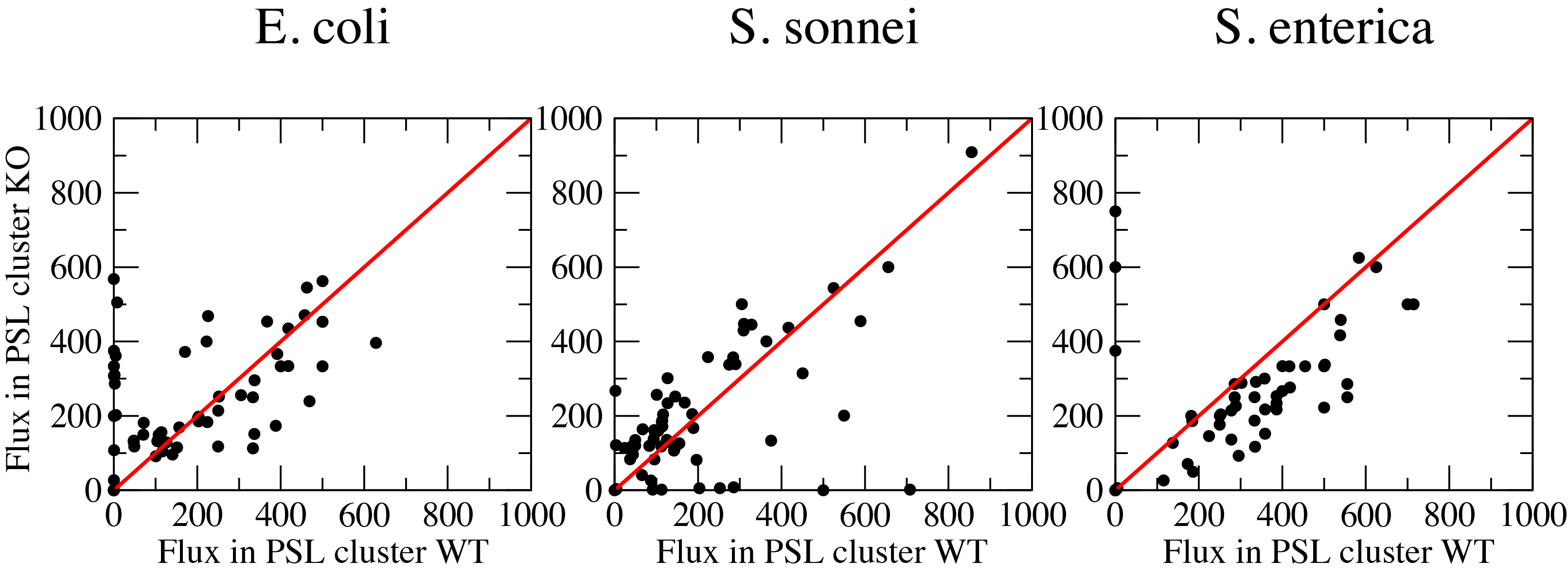

Supplement: S2 Fig — Scatter plots in which each dot represents a PSL cluster and the diagonal line denotes equal average fluxes. (TIF) [file pcbi.1005949.s006.tif]

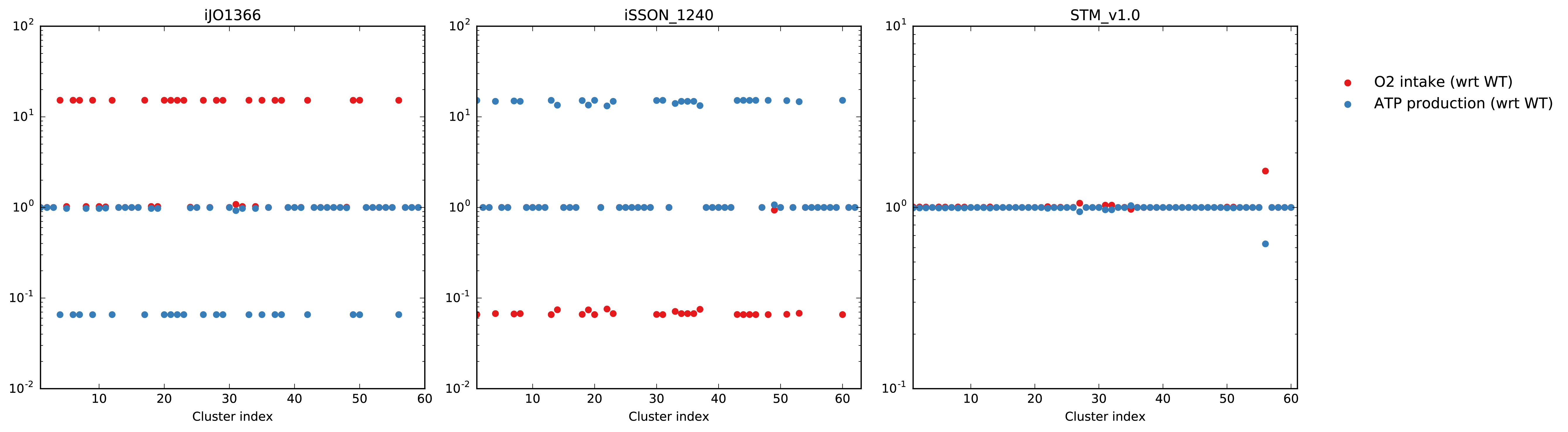

Supplement: S3 Fig — Each dot represents a PSL cluster. (TIF) [file pcbi.1005949.s007.tif]

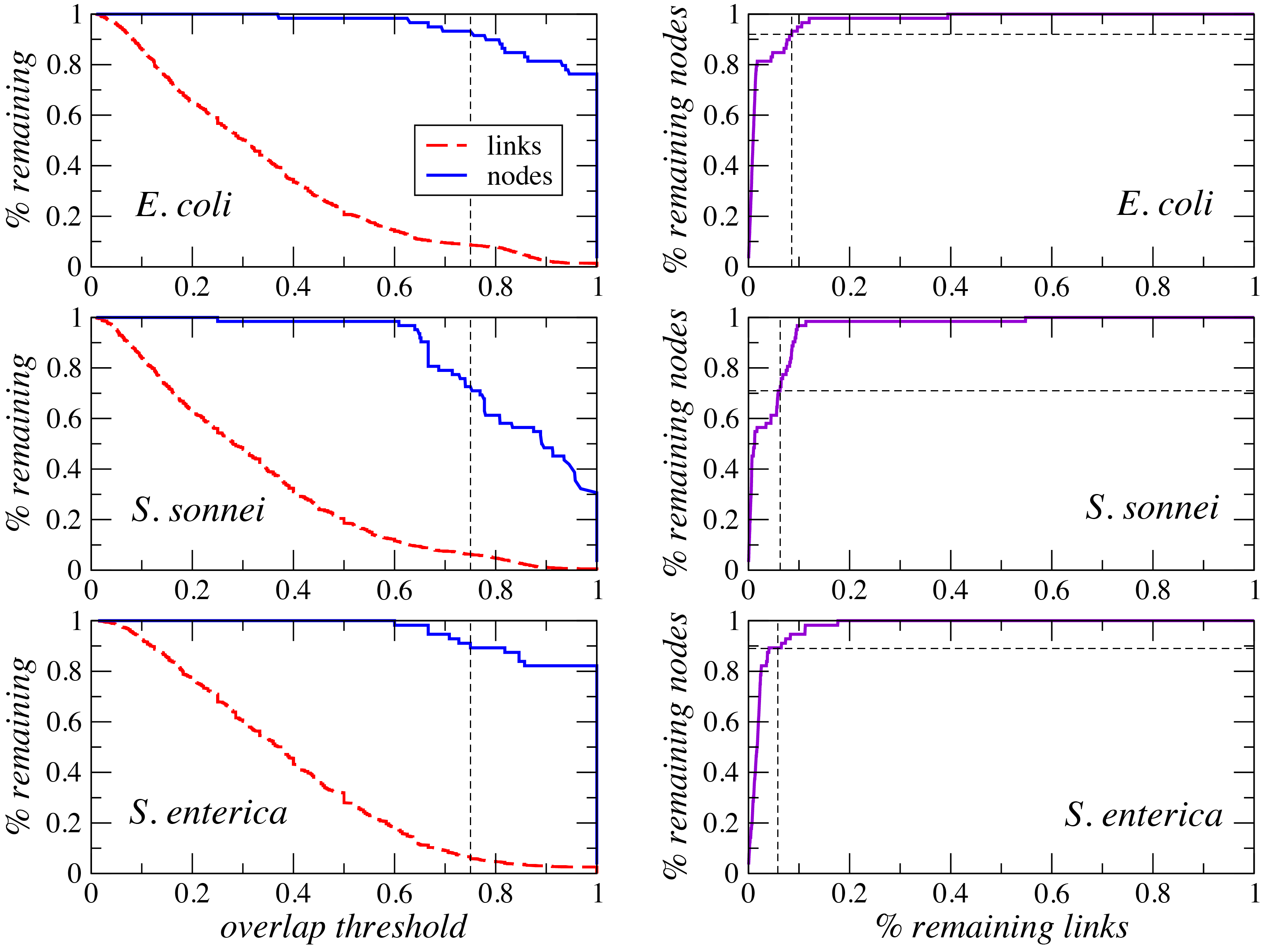

Supplement: S4 Fig — In a backbone, two PSL clusters are connected by a directed link from PSL cluster i to PSL cluster j if more than a certain fraction of reactions in i, given by the overlap threshold, are also in j. Left column: number of clusters and links remaining in the backbone as a function of the threshold. Right columns: fraction of clusters as a function of the fraction of links remaining in the backbone as the threshold is varied. Dashed lines mark the threshold used in the main text and in the next figure. Notice that, for the three bacteria, the region around 75% for the overlap threshold gives a good compromise between preserving the maximum number of clusters in the backbone and reducing the number of links to the minimum. (TIF) [file pcbi.1005949.s008.tif]

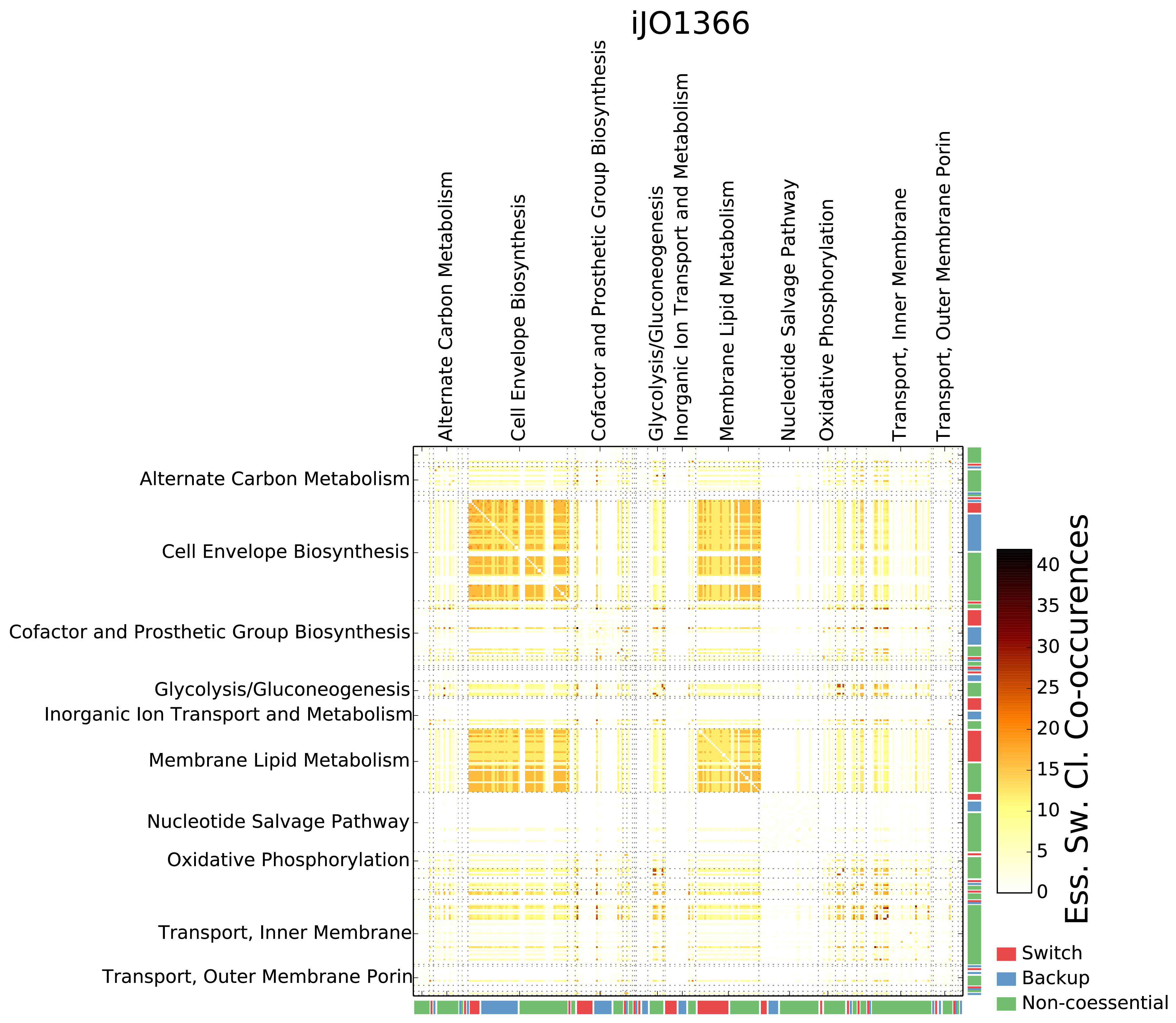

Supplement: S5 Fig — We annotated reactions in PSL pairs in terms of biochemical pathways to provide a network representation where pathways are nodes linked whenever they participate together in a PSL interaction. The network summarizes which pathways are, overall, a metabolic backup of others, for the case of (a) E. coli, (b) S. sonnei and (c) S. enterica. We observe that PSL pairs happen mostly intra-pathway, with the striking exception of the strong entanglement between Cell Envelope Biosysthesis and Membrane Lipid Metabolism in E. coli and S. sonnei. (TIF) [file pcbi.1005949.s009.tif]

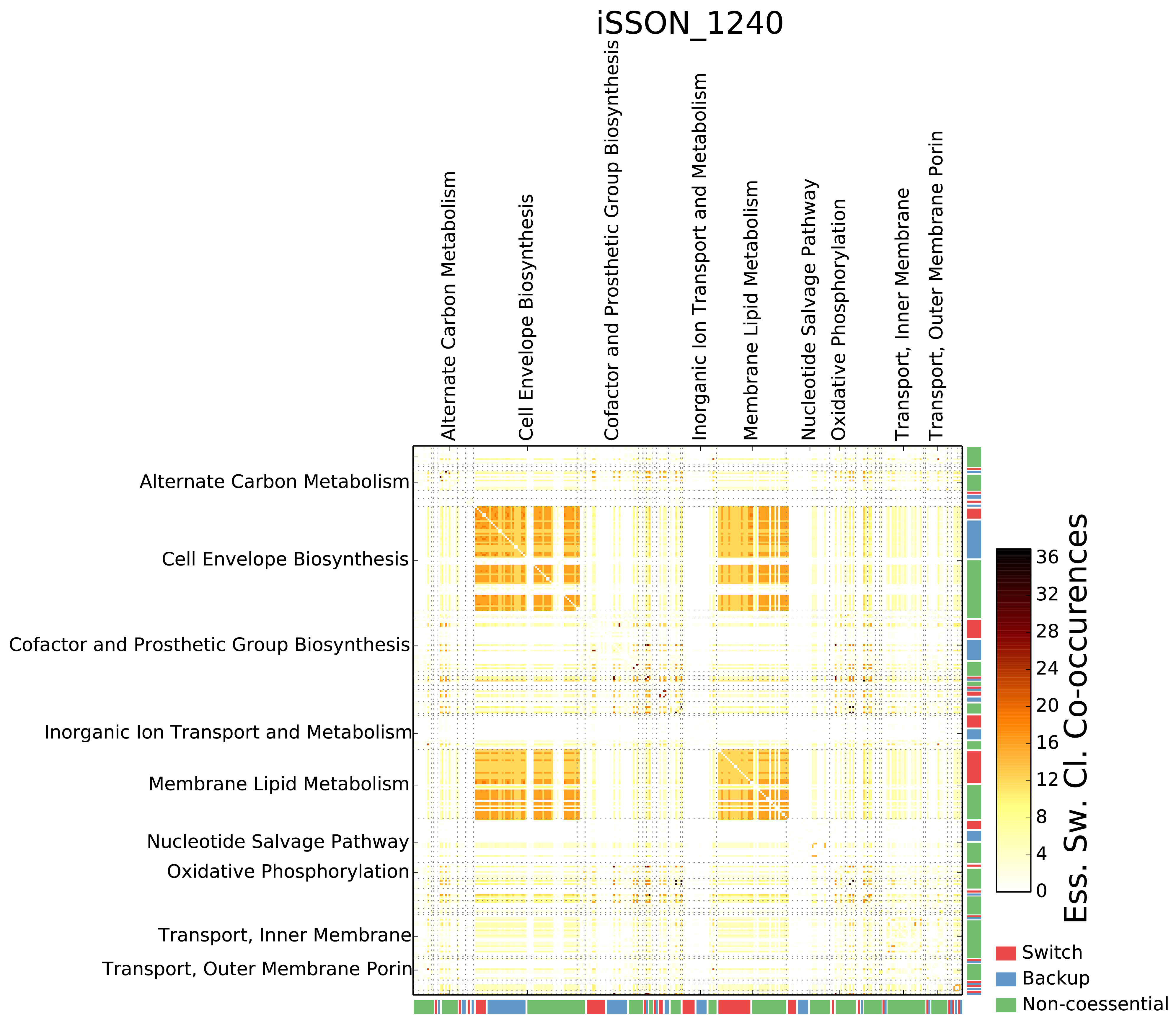

Supplement: S6 Fig — Each matrix shows the number of clusters in which a pair of reactions in PSL clusters coappear. Reactions are ordered according to their metabolic pathway. Pathways are numbered and reported in the list below the matrices. The type of reactions (switch, backup, noncoessential) in the pair is denoted by the color key besides the matrices, e.g. switch reactions are in red. Each entry in the matrix corresponds to the number of PSL clusters in which the corresponding pair of reactions coappear. (TIF) [file pcbi.1005949.s010.tif]

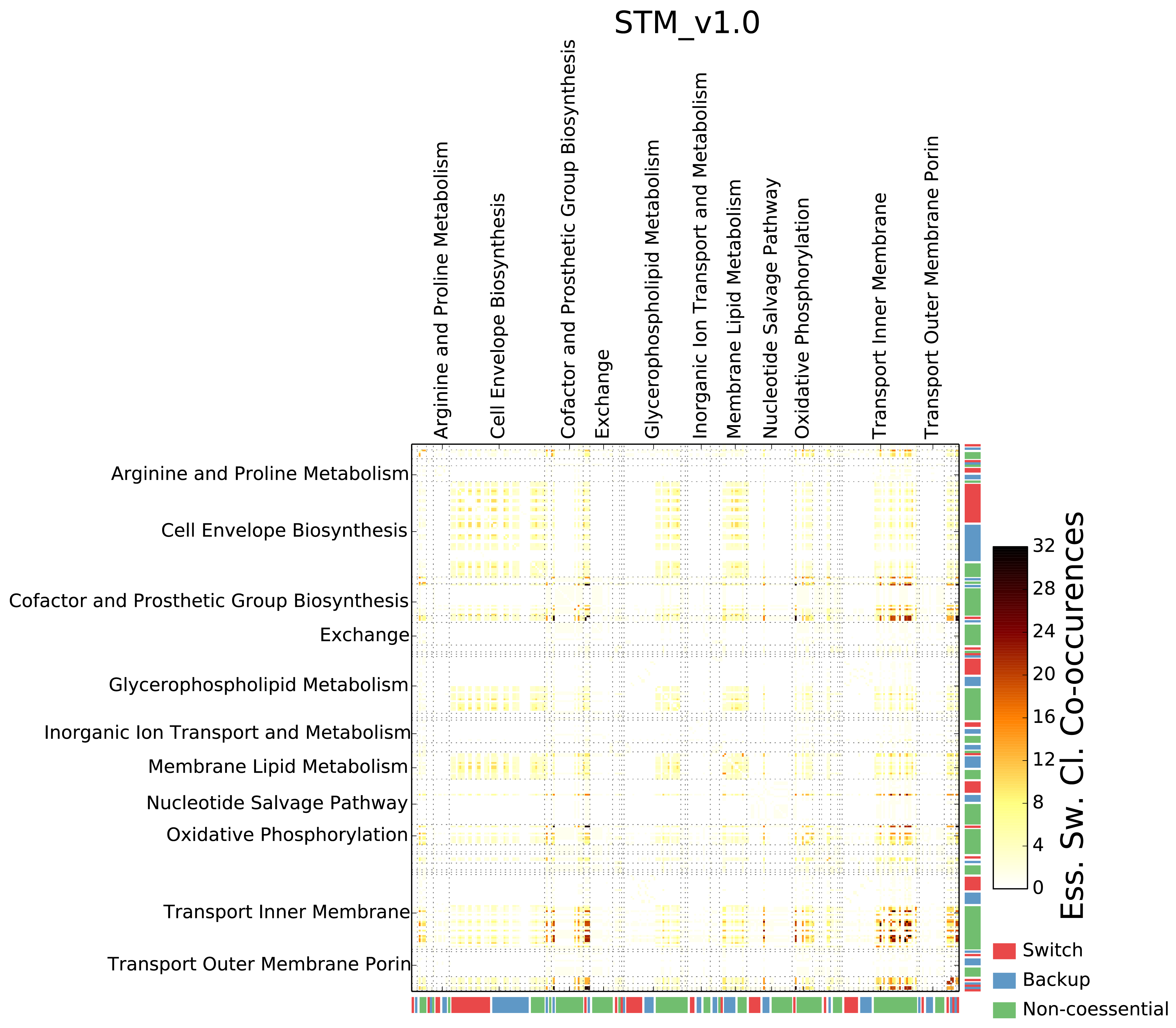

Supplement: S7 Fig — Each matrix shows the number of clusters in which a pair of reactions in PSL clusters coappear. Reactions are ordered according to their metabolic pathway. Pathways are numbered and reported in the list below the matrices. The type of reactions (switch, backup, noncoessential) in the pair is denoted by the color key besides the matrices, e.g. switch reactions are in red. Each entry in the matrix corresponds to the number of PSL clusters in which the corresponding pair of reactions coappear. (TIF) [file pcbi.1005949.s011.tif]

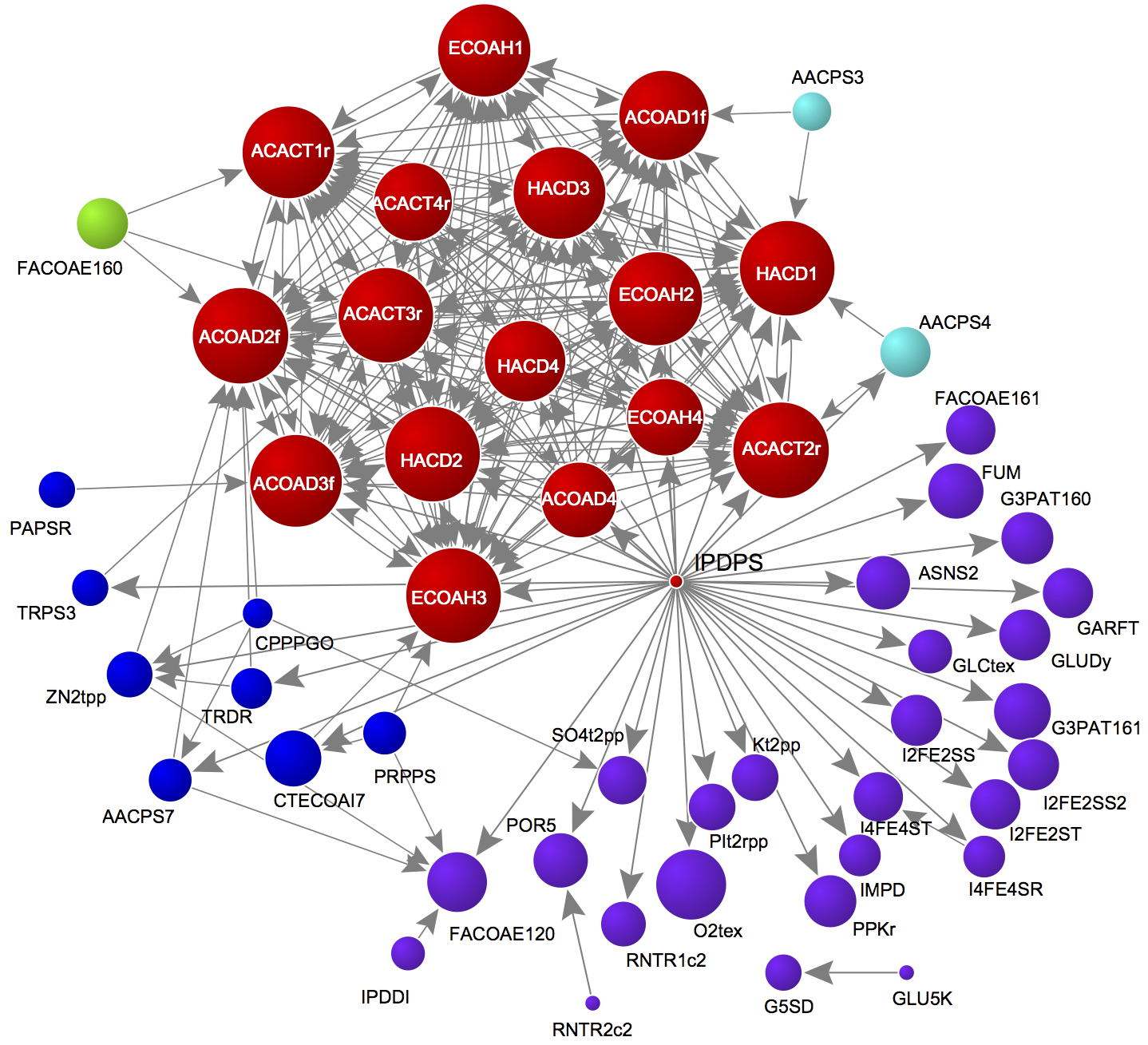

Supplement: S8 Fig — Each matrix shows the number of clusters in which a pair of reactions in PSL clusters coappear. Reactions are ordered according to their metabolic pathway. Pathways are numbered and reported in the list below the matrices. The type of reactions (switch, backup, noncoessential) in the pair is denoted by the color key besides the matrices, e.g. switch reactions are in red. Each entry in the matrix corresponds to the number of PSL clusters in which the corresponding pair of reactions coappear. (TIF) [file pcbi.1005949.s012.tif]

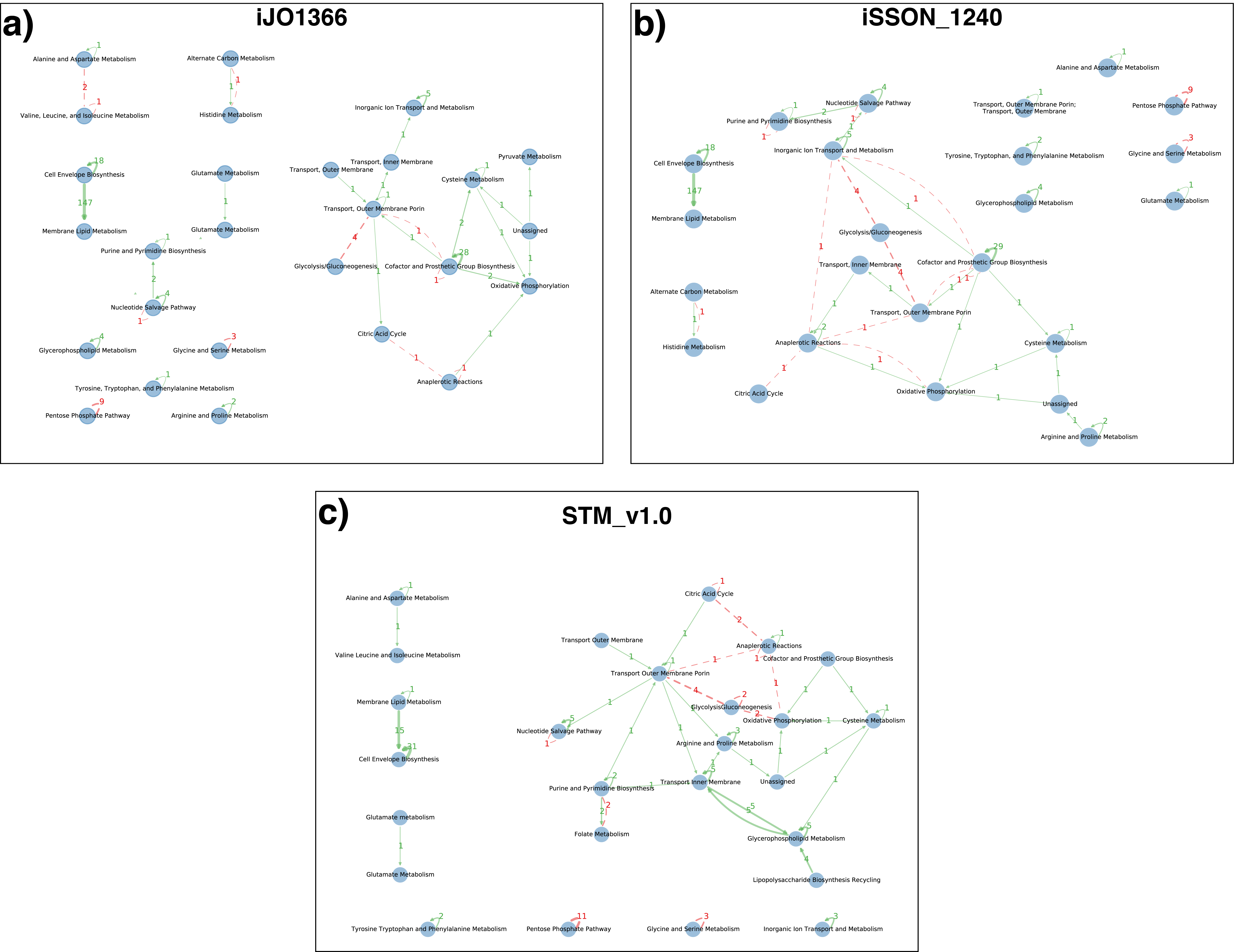

Supplement: S9 Fig — The graph is obtained by considering that two PSL clusters are connected by a directed link from PSL cluster i to PSL cluster j if at least 75% of the reactions in i are also in j. Nodes represent PSL clusters. The size of a node is proportional to the size of the corresponding PSL cluster in number of reactions and the color indicates its maximum k-core out, where a k-core out k − c is defined as the maximal subgraph of PSL clusters such that all the PSL clusters in it have at least k − c outgoing connections inside the subgraph. (TIFF) [file pcbi.1005949.s013.tiff]

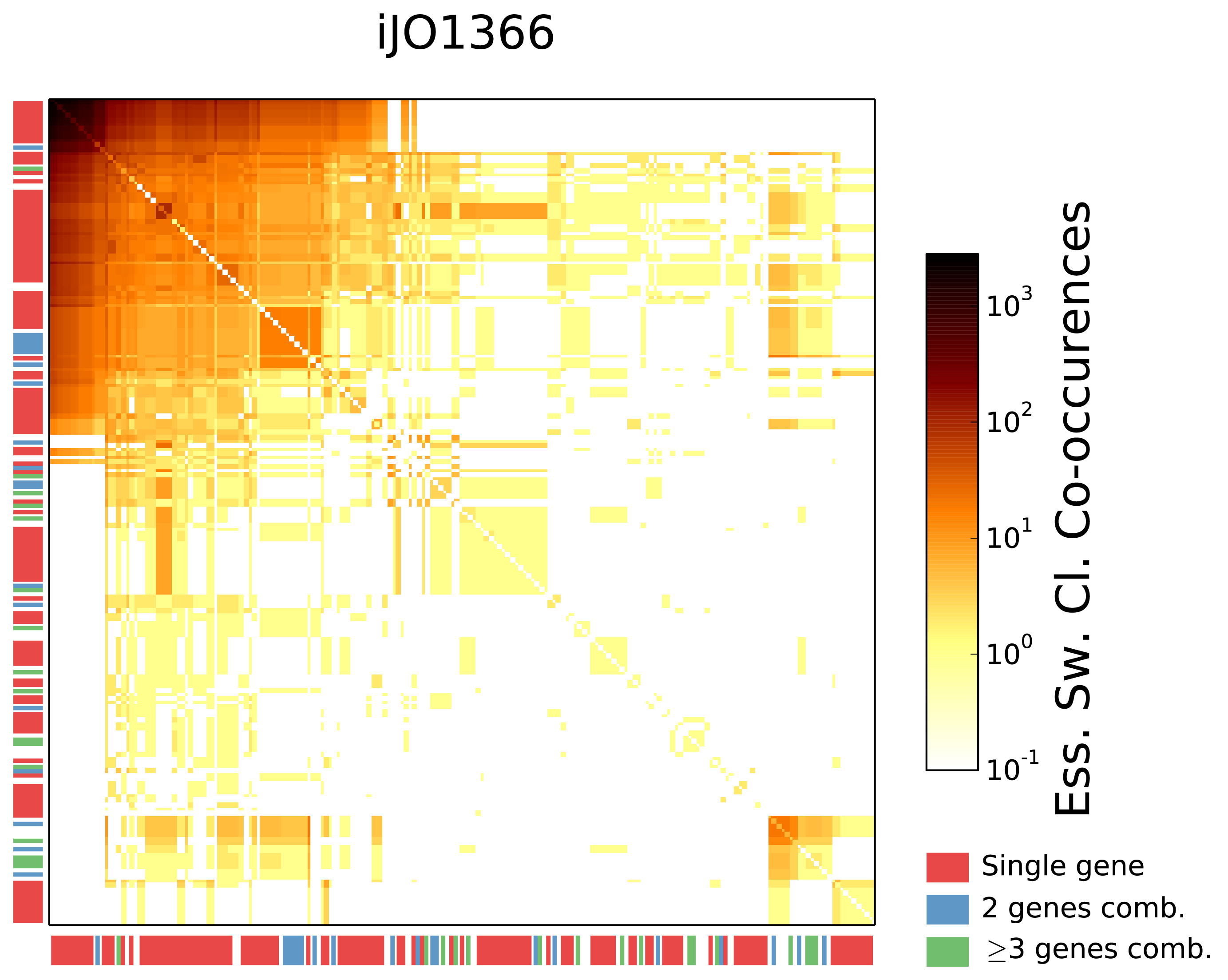

Supplement: S10 Fig — Each matrix shows the number of sets in which a pair of genetic units (gene or gene complexes) in PSL clusters coappear. Each complex is composed of a number of genes varying from 1 up to 13 and may appear more than once in each set. For this reason, pairs of gene complexes may have a cooccurrence frequency that exceeds the number of sets, as it can be observed mostly in the upper diagonal part of the matrices. The number of genes in the complex is denoted by the color key beside the matrix (e.g. red denotes single genes). (TIF) [file pcbi.1005949.s014.tif]

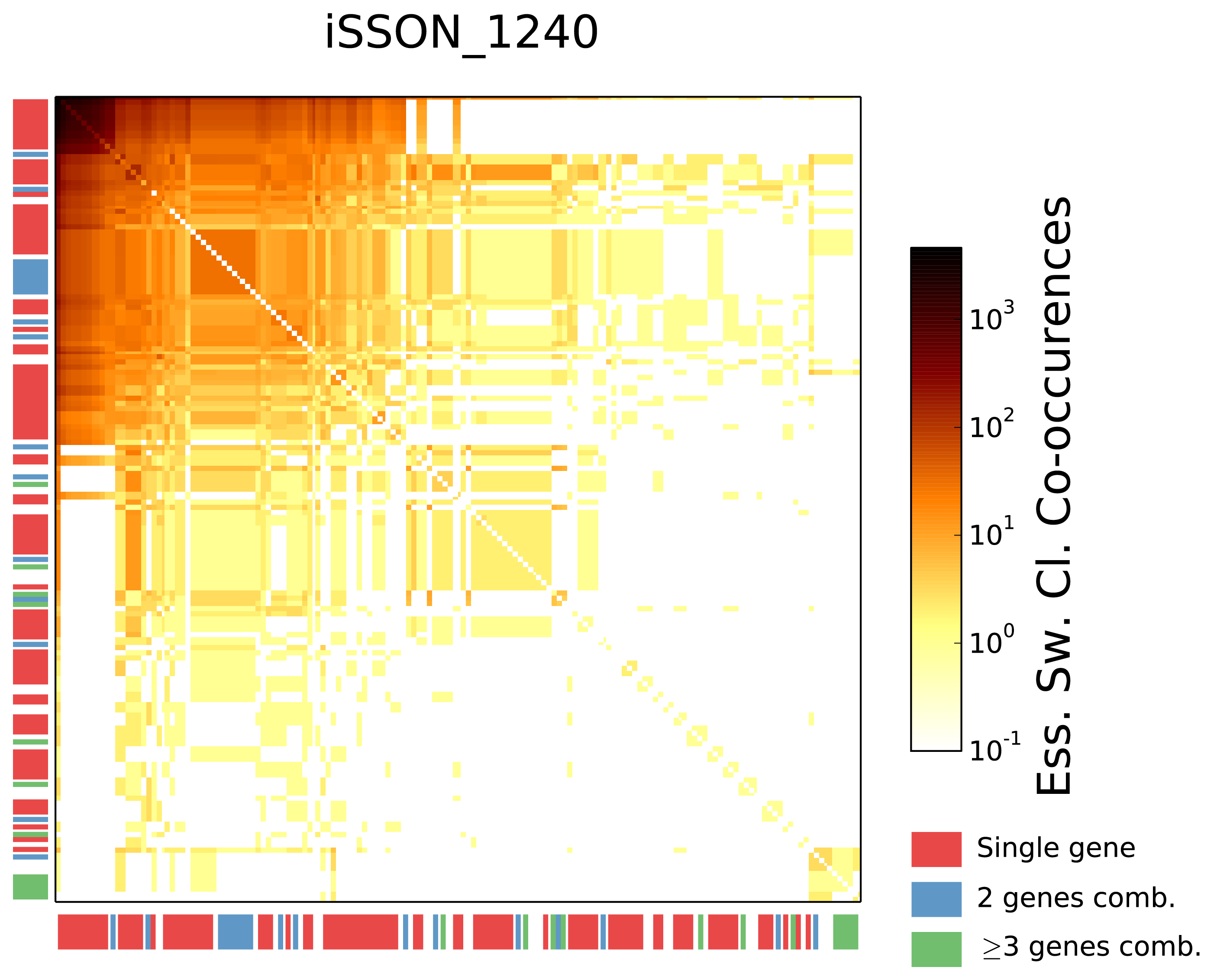

Supplement: S11 Fig — Each matrix shows the number of sets in which a pair of genetic units (gene or gene complexes) in PSL clusters coappear. Each complex is composed of a number of genes varying from 1 up to 13 and may appear more than once in each set. For this reason, pairs of gene complexes may have a cooccurrence frequency that exceeds the number of sets, as it can be observed mostly in the upper diagonal part of the matrices. The number of genes in the complex is denoted by the color key beside the matrix (e.g. red denotes single genes). (TIF) [file pcbi.1005949.s015.tif]

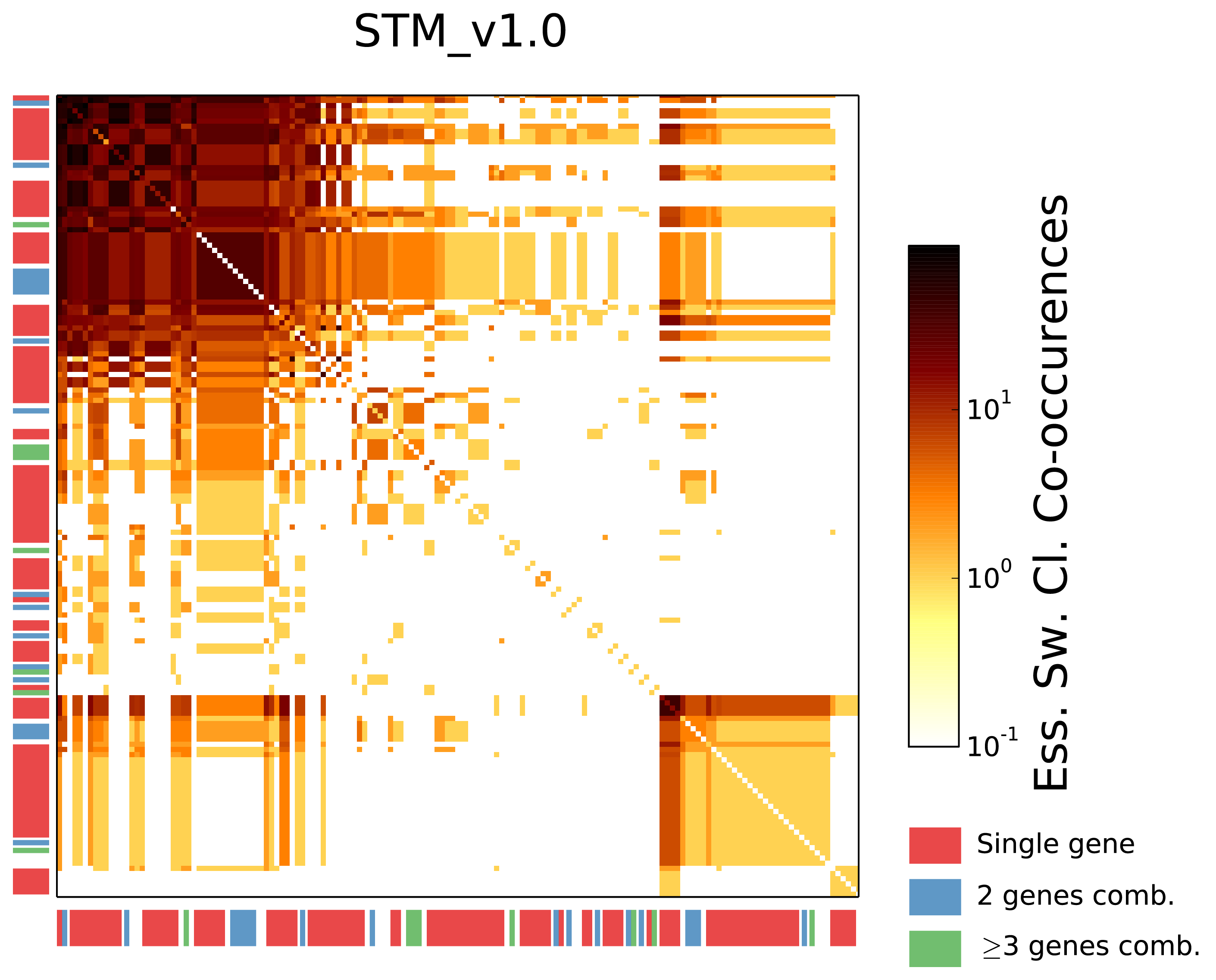

Supplement: S12 Fig — Each matrix shows the number of sets in which a pair of genetic units (gene or gene complexes) in PSL clusters coappear. Each complex is composed of a number of genes varying from 1 up to 13 and may appear more than once in each set. For this reason, pairs of gene complexes may have a cooccurrence frequency that exceeds the number of sets, as it can be observed mostly in the upper diagonal part of the matrices. The number of genes in the complex is denoted by the color key beside the matrix (e.g. red denotes single genes). (TIF) [file pcbi.1005949.s016.tif]
